# Supplementary material for: Montmorillonite Catalyzed Synthesis of Novel Steroid Dimers
Source: Molecules. 2023 Oct 13;28(20):7068. doi: 10.3390/molecules28207068 (PMC10609449; doi:10.3390/molecules28207068)

Supporting Information

Montmorillonite catalyzed synthesis of novel steroid dimers

Aneta M. Tomkiel,[1,*] Adam D. Majewski,[1,2] Leszek Siergiejczyk,[1] Jacek W. Morzycki[1,*]

[1] Faculty of Chemistry, University of Bialystok, Ciołkowskiego 1K, 15-245 Białystok, Poland.

[2] Doctoral School of Exact and Natural Sciences at the University of Bialystok, Ciołkowskiego 1K, 15-245 Białystok, Poland

E-mail: morzycki.uwb.edu.pl, a.tomkiel@uwb.edu.pl

Contents:

1. General experimental data (SI-2)

2. General experimental procedure for the formation of all new compounds (SI-2 – SI-4)

3. Compound characterization data (SI-4 – SI-7)

4. Assignments of ^{1}H and ^{13}C NMR signals for compounds **11a** and **11b** (SI-8)

5. References (SI-8)

6. ^{1}H and ^{13}C NMR spectra of all new compounds (SI-9 – SI-24)

1. General experimental data

The reagents were purchased from Merck, Alfa Aesar, or Acros. All solvents were freshly distilled prior to use. Anhydrous solvents were prepared by distillation over appropriate drying agents under an argon atmosphere.

The reactions were monitored by TLC on silica gel plates 60 F254 (Merck, Darmstadt, Germany), and spots were visualized either by a UV hand lamp (Type: NU-4;254 nm/365 nm, 2x4W, Herolab GmbH Laborgeräte, Wiesloch, Germany) or by charring with molybdophosphoric acid/cerium(IV) sulfate in H_2SO_4. The reaction products were isolated by chromatographic methods using JT Baker silica gel (J.T. Baker, Phillipsburg, NJ, USA), pore size 40 Å (70–230 mesh).

^{1}H and ^{13}C NMR (400 and 100 MHz, respectively) spectra of all compounds were recorded using a Bruker Avance II spectrometer (Bruker, Fällanden, Switzerland) in a $CDCl_3$ or $CDCl_3$/MeOD mixture and referenced to TMS (0.0 ppm) and $CDCl_3$ (77.0 ppm), respectively. Only selected signals in the ^{1}H NMR spectra are reported. The original ^{1}H and ^{13}C NMR spectra are contained in the Supplementary Materials. Infrared spectra were recorded using Attenuated Total Reflectance (ATR) as solid samples with a Nicolet 6700 FT-IR spectrometer (Thermo Fisher Scientific, Waltham, MA, USA). Mass spectra were obtained at the Accurate-Mass Q-TOFLC/MS 6530 spectrometer (Agilent, Santa Clara, NJ, USA) with electrospray ionization (ESI). Melting points were determined on a Kofler bench (Boetius type, Nagema, VEB Wägetechnik Rapido Radebeul, Dresden, Germany) melting point apparatus.

Tosylates **4b** [1] and **6b** [2] were prepared according to literature procedures.

Unmodified montmorillonite K10 or metal cation-exchanged montmorillonites: H^+-K10, Ti^{4+}-K10 and Cu^{2+}-K10 were used as the catalyst. Modified forms: H^+-K10 [3], Ti^{4+}-K10 [4] and Cu^{2+}-K10 [5] was obtained according to the literature procedures. All catalysts were activated prior to use by calcination at high temperatures (unmodified montmorillonite K10 at 120 °C, 200 °C, 280 °C, 400 °C, or 500 °C; H^+-K10 at 280 °C; Ti^{4+}-K10 at 280 °C or 500 °C; and Cu^{2+}-K10 at 120 °C.

Chloroform was usually used as the solvent for the Montmorillonite K10-catalyzed reaction. Before use, it was purified from the stabilizer (ethanol) contained in a commercially available solvent. When chloroform with a stabilizer was used, steroidal ethyl ether (17%) as an undesirable by-product was formed. Refluxed 1,2-dichloroethane, cyclohexane, toluene, xylenes, acetonitrile, acetone, THF, and dioxane as solvents were also tested.

Some of the products obtained are described in the literature: diosgenin-derived disteroidal ether [6], dicholesteryl ether, hydroquinone dicholesteryl ether, and hydroquinone mono cholesteryl ether [7].

2. General experimental procedure for the formation of all new compounds

*Solvent-free reaction of tosylate **4b** with hydroquinone*

The mixture of tosylate **4b** (100 mg. 0.2 mmol) and hydroquinone (11 mg. 0.1 mmol) was stirred and heated to 120 °C for 5 minutes under an argon atmosphere. Then, the reaction mixture was cooled to room temperature and subjected to column chromatography on silica gel, which resulted in the separation of compounds: **7** and **8** (were eluted with hexane in 23% yield), **11a** (was eluted with hexane/ethyl acetate 97:3 mixture in 7% yield), **11c** (was eluted with hexane/ethyl acetate 95:5 mixture in 9% yield), **10a** (was eluted with hexane/ethyl acetate 93:7 mixture in <5% yield), **11b** (was eluted with hexane/ethyl acetate 93:7 mixture in 5% yield), **9** (was eluted with hexane/ethyl acetate

87:13 mixture in 12% yield), and **10b** (was eluted with hexane/ethyl acetate 87:13 mixture in <5% yield).

*Optimal procedure for preparation of dimer **1** in the montmorillonite K10 catalyzed reaction of androst-5-en-3β-ol-17-one (**4a**) with hydroquinone*

A mixture of androst-5-en-3β-ol-17-one (**4a**) (100 mg, 0.35 mmol), hydroquinone (15.4 mg, 0.14 mmol), unmodified montmorillonite K10 activated at 280 °C (500 mg), and dry $CHCl_3$ (5 mL) was refluxed under argon conditions and monitored by TLC. After completion of the reaction (4 hours), the suspension was filtered through a sintered glass funnel, and the precipitate was washed with a methanol/chloroform (2:8) mixture (3 x 50 mL). The filtrate was evaporated in a vacuum. The residue was subjected to column chromatography on silica gel, which resulted in the separation of compounds **7** and **8** (were eluted with hexane in 28% yield), **12** (was eluted with hexane/ethyl acetate 95:5 mixture in 7% yield), **1** (was eluted with hexane/ethyl acetate 94:6 mixture in 23% yield), **9** (was eluted with hexane/ethyl acetate 87:13 mixture in 12% yield), and small amounts of compounds **11a**, **11b,** and **11c** (total <5%).

Analogously, steroid dimers **2** (12%) and **3** (28%) were obtained from diosgenin (**5a**) and cholesterol (**6a**), respectively, according to the procedure described above for androst-5-en-3β-ol-17-one (**4a**). Undesirable elimination products (~35% in both cases), disteroid ethers (12% and 22%, respectively) and mono ethers (12% and 10%, respectively), were also formed.

*Optimal procedure for preparation of dimer **12** in the montmorillonite K10-catalyzed reaction of androst-5-en-3β-ol-17-one (**4a**)*

A mixture of androst-5-en-3β-ol-17-one (**4a**) (100 mg, 0.35 mmol), hydroquinone (15.4 mg, 0.14 mmol), modified montmorillonite H^+-K10 activated at 280 °C (500 mg), and dry $CHCl_3$ (5 mL) was heated to 50 °C for 24 hours under argon conditions and monitored by TLC. After completion of the reaction, the suspension was filtered through a sintered glass funnel, and the precipitate was washed with a methanol/chloroform 2:8 mixture (3 x 50 mL). The filtrate was evaporated in a vacuum. The residue was subjected to column chromatography on silica gel, which resulted in the separation of compounds **7** and **8** (eluted with hexane in 13% yield), **12** (eluted with hexane/ethyl acetate 95:5 mixture in 57% yield), **1** (eluted with hexane/ethyl acetate 94:6 mixture in 1% yield), and **9** (eluted with hexane/ethyl acetate 87:13 mixture in 7% yield).

*Optimal procedure for preparation of dimer **1** in the montmorillonite K10-catalyzed reaction of tosylate **4b** with hydroquinone*

A mixture of tosylate **4b** (88.5 mg; 0.2 mmol), hydroquinone (8.4 mg; 0.076 mmol), modified montmorillonite Ti^{4+}-K10 activated at 280 °C (300 mg), and dry $CHCl_3$ (5 mL) was mixed under argon conditions and monitored by TLC. After completion of the reaction (3 days), the suspension was filtered through a sintered glass funnel, and the precipitate was washed with a methanol/chloroform (2:8) mixture (3 x 50 mL). The filtrate was evaporated in a vacuum. The residue was subjected to column chromatography on silica gel, which resulted in the separation of compounds **7** and **8** (eluted with hexane in 28% yield), **1** (eluted with hexane/ethyl acetate 94:6 mixture in 31% yield), and **9** (eluted with hexane/ethyl acetate 87:13 mixture in 19% yield).

*Optimal procedure for preparation of dimer **3** in the montmorillonite K10-catalyzed reaction of tosylate **6b** with hydroquinone*

A mixture of tosylate **6b** (100 mg; 0.2 mmol), hydroquinone (8.4 mg; 0.076 mmol), unmodified montmorillonite K10 activated at 500 °C (50 mg), and dry $CHCl_3$ (5 mL) was mixed under argon conditions and monitored by TLC. After completion of the reaction (3 days), the suspension was filtered through a sintered glass funnel, and the precipitate was washed with a methanol/chloroform (2:8) mixture (3 x 50 mL). The filtrate was evaporated in a vacuum. The residue was subjected to column chromatography on silica gel, which resulted in the separation of elimination products (eluted with hexane in 10% yield) and **3** (eluted with hexane/ethyl acetate in a 96:4 mixture in 67% yield).

3. Compounds characterization data

Compound 1

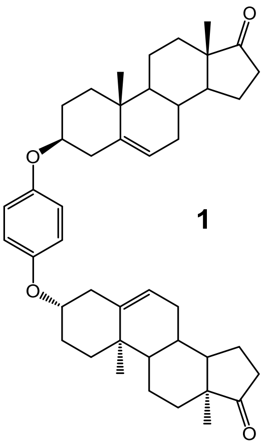

1

Colorless crystals (CH_2Cl_2/ethyl acetate); mp 252–254 °C; Rf = 0.50 (3 x benzene/ethyl acetate 94:6); IR (ATR) ν_{max} 2938, 2907, 1744, 1731, 1501, 1214, 1043, 1029, 815 cm^{-1}; ^{1}H NMR ($CDCl_3$, 400 MHz) δ 6.82 (4H, s, H-Ar), 5.42 (2H, m, H-6), 4.00 (2H, m, H-3α), 1.09 (6H, s, H-19), 0.91 (6H, s, H-18); ^{13}C NMR ($CDCl_3$, 100 MHz) δ 221.0 (2 x C), 151.8 (2 x C), 140.7 (2 x C), 121.4 (2 x CH), 117.4 (4 x CH), 77.9 (2 x CH), 51.8 (2 x CH), 50.3 (2 x CH), 47.5 (2 x C), 38.8 (2 x CH_2), 37.1 (2 x CH_2), 37.0 (2 x C), 35.8 (2 x CH_2), 31.5 (2 x CH), 31.4 (2 x CH_2), 30.8 (2 x CH_2), 28.3 (2 x CH_2), 21.9 (2 x CH_2), 20.4 (2 x CH_2), 19.4 (2 x CH_3), 13.5 (2 x CH_3); HRMS *m/z* 651.4409 (calcd for $C_{44}H_{59}O_4^+$, 651.4408).

Compound 2

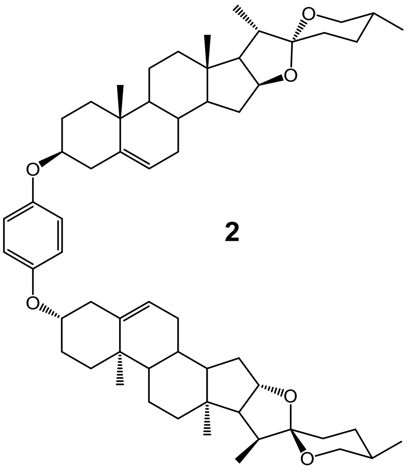

2

Colorless crystals (hexane/CH_2Cl_2); mp 307–309 °C; Rf = 0.38 (3 x benzene/ethyl acetate 94:6); IR (ATR) ν_{max} 2925, 1502, 1225, 1050, 1016, 809 cm^{-1}; ^{1}H NMR ($CDCl_3$, 400 MHz) δ 6.82 (4H, s, H-Ar), 5.38 (2H, m, H-6), 4.43 (2H, m, H-16), 3.98 (2H, m, H-3α), 3.49 (2H, m, H-26β), 3.39 (2H, t, *J* = 10.9 Hz, H-26α), 1.08 (6H, s, H-19), 0.99 (6H, d, *J* = 6.9 Hz, H-21), 0.81 (6H, s, H-18), 0.80 (6H, d, *J* = 5.0 Hz, H-27); ^{13}C NMR ($CDCl_3$, 100 MHz) δ 151.8 (2 x C), 140.5 (2 x C), 121.9 (2 x CH), 117.4 (4 x CH), 109.3 (2 x C), 80.8 (2 x CH), 78.0 (2 x CH), 66.8 (2 x CH_2), 62.1 (2 x CH), 56.5 (2 x CH), 50.1 (2 x CH), 41.6 (2 x CH), 40.3 (2 x C), 39.8 (2 x CH_2), 38.8 (2 x CH_2), 37.2 (2 x CH_2), 37.0 (2 x C), 32.1 (2 x CH_2), 31.8 (2 x CH_2), 31.43 (2 x CH), 31.38 (2 x CH_2), 30.3 (2 x CH), 28.8 (2 x CH_2), 28.3 (2 x CH_2), 20.8 (2 x CH_2), 19.4 (2 x CH_3), 17.1 (2 x CH_3), 16.3 (2 x CH_3), 14.5 (2 x CH_3); HRMS *m/z* 903.6502 (calcd for $C_{60}H_{87}O_6^+$, 903.6497).

Compound 3 [7]

Colorless crystals (hexane/CH_2Cl_2); mp 223-226 °C; Rf = 0.41 (hexane/ ethyl acetate 9.5:0.5); IR ν_{max}: 1602, 1503, 1033, 1018, 830; ^{1}H NMR ($CDCl_3$, 400 MHz) δ 6.82 (4H, s, H-Ar), 5.38 (2H, m, H-6), 3.99 (2H, m, H-3α), 1.06 (6H, s, H-19), 0.93 (6H, d, *J* = 6.5 Hz, H-21), 0.884 (6H, d, *J* = 6.6 Hz, H-26 or H-27), 0.879 (6H, d, *J* = 6.6 Hz, H-26 or H-27), 0.70 (3H, s, H-18); ^{13}C NMR ($CDCl_3$, 100 MHz) δ 151.9 (2 x C), 140.5 (2 x C), 122.1 (2 x CH), 117.4 (2 x CH), 78.2 (2 x CH), 56.8 (2 x CH), 56.2 (2 x CH), 50.2 (2 x CH), 42.3 (2 x C), 39.8 (2 x CH_2), 39.5 (2 x CH_2), 38.9 (2 x CH_2), 37.2 (2 x CH_2), 36.9 (2 x C), 36.2 (2 x CH_2), 35.8 (2 x CH), 32.0 (2 x CH_2), 31.9 (2 x CH), 28.4 (2 x CH_2), 28.2 (2 x CH_2), 28.0 (2 x CH), 24.3 (2 x CH_2), 23.8 (2 x CH_2), 22.8 (2 x CH_3), 22.6 (2 x CH_3), 21.1 (2 x CH_2), 19.4 (2 x CH_3), 18.7 (2 x CH_3), 11.9 (2 x CH_3); EI MS, m/z: 846 (M+, 3%), 369 [(cholest-5-en-3-yl)+, 100%].

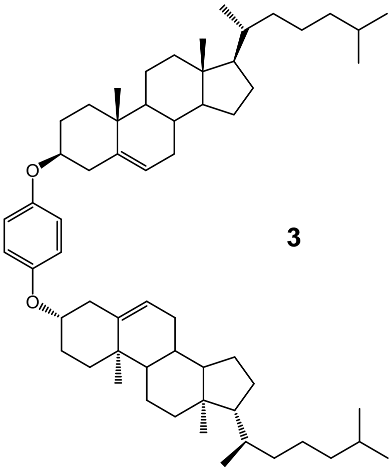

3

Compound 7

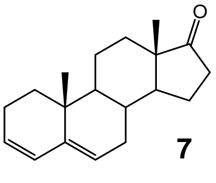

7

White solid (hexane/CH_2Cl_2); mp 85–87 °C; Rf = 0.32 (hexane/ethyl acetate 9:1); IR (ATR) ν_{max} 2912, 2856, 1739 cm^{-1}; ^{1}H NMR ($CDCl_3$, 400 MHz) δ 5.95 (1H, d, *J* = 9.9 Hz, H-4), 5.63 (1H, m, H-3), 5.42 (1H, m, H-6), 2.48 (1H, dd, *J* = 19.2 Hz, *J* = 8.8 Hz, H-16β), 0.99 (3H, s, H-19), 0.93 (3H, s, H-18); ^{13}C NMR ($CDCl_3$, 100 MHz) δ 221.1 (C), 141.6 (C), 128.7 (CH), 125.4 (CH), 122.1 (CH), 51.9 (CH), 48.5 (CH), 47.7 (C), 35.8 (CH_2), 35.3 (C), 33.7 (CH_2), 31.44 (CH_2), 31.41 (CH), 30.6 (CH_2), 23.0 (CH_2), 21.8 (CH_2), 20.3 (CH_2), 18.8 (CH_3), 13.7 (CH_3); HRMS *m/z* 271.2054 (calcd for $C_{19}H_{27}O^+$, 271.2056).

Compound 8

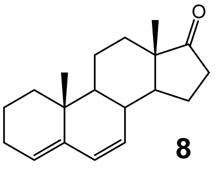

8

White solid; Rf = 0.36 (hexane/ethyl acetate 9:1); IR (ATR) ν_{max} 2916, 1736, 1506 cm^{-1}; ^{1}H NMR ($CDCl_3$, 400 MHz) δ 5.99 (1H, dd, J = 9.8 Hz, J = 2.5 Hz, H-4), 5.56 (1H, d, J = 9.8 Hz, H-7), 5.47 (1H, m, H-3), 0.98 (3H, s, H-19), 0.96 (3H, s, H-18); ^{13}C NMR ($CDCl_3$, 100 MHz) δ 220.7 (C), 142.2 (C), 129.9 (CH), 125.6 (CH), 124.7 (CH), 51.6 (CH), 49.6 (CH), 48.3 (C), 36.5 (CH), 35.8 (CH_2), 34.8 (C), 34.5 (CH_2), 31.6 (CH_2), 25.3 (CH_2), 21.5 (CH_2), 20.2 (CH_2), 18.4 (CH_2), 18.3 (CH_3), 13.8 (CH_3); HRMS *m/z* 271.2061 (calcd for $C_{19}H_{27}O^+$, 271.2056).

Compound 9

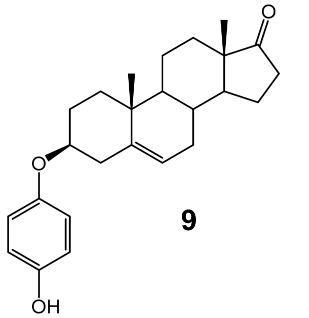

9

Colorless crystals (CH_2Cl_2/ethyl acetate); mp 277–279 °C; Rf = 0.48 (3 x hexane/ethyl acetate 74:26); IR (ATR) ν_{max} 3311, 2948, 2864, 1710, 1505, 1211, 1028, 819 cm^{-1}; ^1H NMR ($CDCl_3$/MeOD, 400 MHz) δ 6.76 (2H, d, *J* = 19.2 Hz, H-Ar), 6.71 (2H, d, *J* = 19.1 Hz, H-Ar), 5.37 (1H, m, H-6), 3.93 (1H, m, H-3α), 1.05 (3H, s, H-19), 0.87 (3H, s, H-18); ^{13}C NMR ($CDCl_3$/MeOD, 100 MHz) δ 221.9 (C), 150.8 (C), 150.7 (C), 140.7 (C), 121.3 (CH), 117.9 (2 x CH), 115.8 (2 x CH), 78.3 (CH), 51.7 (CH), 50.2 (CH), 47.6 (C), 38.8 (CH_2), 37.0 (CH_2), 36.9 (C), 35.8 (CH_2), 31.4 (CH), 31.3 (CH_2), 30.7 (CH_2), 28.2 (CH_2), 21.8 (CH_2), 20.3 (CH_2), 19.4 (CH_3), 13.5 (CH_3); HRMS *m/z* 381.2438 (calcd for $C_{25}H_{33}O_3^+$, 381.2424).

Compound 10a

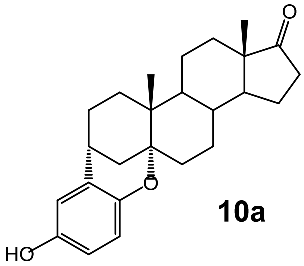

10a

White solid; Rf = 0.66 (3 x hexane/ethyl acetate 74:26); IR (ATR) ν_{max} 3325, 2927, 1737, 1241, 1206, 1190, 1153, 1077, 1053, 813, 785 cm^{-1}; ^1H NMR ($CDCl_3$, 400 MHz) δ 6.67 (1H, d, J = 8.6 Hz, H-Ar), 6.60 (1H, dd, J = 8.6 Hz, J = 3.0 Hz, H-Ar), 6.48 (1H, d, J = 3.0 Hz, H-Ar), 4.26 (1H, bs, -OH), 2.87 (1H, m, H-3β), 2.46 (1H, dd, J = 18.9 Hz, J = 8.3 Hz, H-16β), 1.05 (3H, s, H-19), 0.88 (3H, s, H-18); ^{13}C NMR ($CDCl_3$, 100 MHz) δ 221.4 (C), 150.1 (C), 147.9 (C), 128.0 (C), 115.3 (CH), 114.2 (CH), 113.9 (CH), 78.5 (C), 51.2 (CH), 47.8 (C), 46.2 (CH), 42.1 (C), 35.9 (CH_2), 34.4 (CH), 33.6 (CH_2), 32.7 (CH), 31.8 (CH_2), 31.5 (CH_2), 29.41 (CH_2), 29.36 (CH_2), 24.8 (CH_2), 21.7 (CH_2), 20.0 (CH_2), 16.2 (CH_3), 13.8 (CH_3); HRMS *m/z* 381.2428 (calcd for $C_{25}H_{33}O_3^+$, 381.2424).

Compound 11a

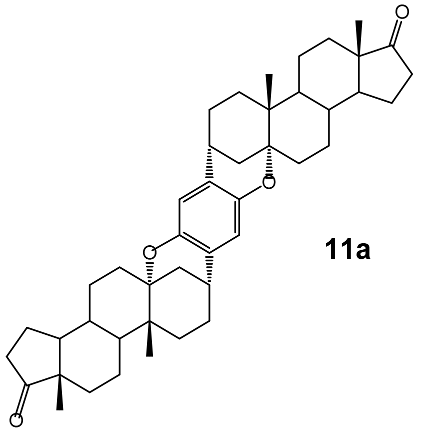

11a

W hite solid; Rf = 0.77 (3 x hexane:ethyl acetate 74/26); IR (ATR) ν_{max} 2915, 2853, 1737, 1242, 1205, 1151, 1001, 813, 785 cm^{-1}; ^1H NMR ($CDCl_3$, 400 MHz) δ 6.41 (2H, s, H-Ar), 2.85 (2H, m, H-3β), 2.46 (2H, dd, *J* = 18.9 Hz, *J* = 8.2 Hz, H-16β), 1.04 (6H, s, H-19), 0.88 (6H, s, H-18); ^{13}C NMR ($CDCl_3$, 100 MHz) δ 221.5 (2 x C), 148.4 (2 x C), 126.1 (2 x C), 112.8 (2 x CH), 78.1 (2 x C), 51.2 (2 x CH), 47.8 (2 x C), 46.1 (2 x CH), 42.1 (2 x C), 35.9 (2 x CH_2), 34.4 (2 x CH), 33.7 (2 x CH_2), 32.6 (2 x CH), 32.2 (2 x CH_2), 31.6 (2 x CH_2), 29.5 (2 x CH_2), 29.3 (2 x CH_2), 24.8 (2 x CH_2), 21.7 (2 x CH_2), 20.0 (2 x CH_2), 16.1 (2 x CH_3), 13.8 (2 x CH_3); HRMS *m/z* 651.4408 (calcd for $C_{44}H_{59}O_4^+$, 651.4408).

Compound 11b

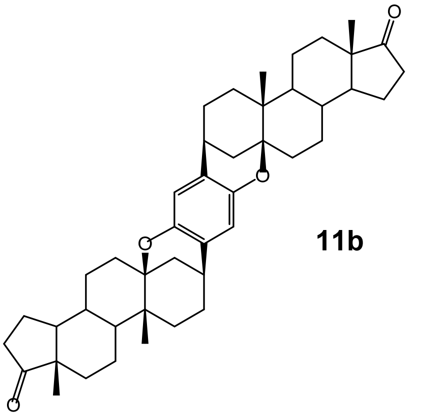

11b

White solid; Rf = 0.66 (3 x hexane/ethyl acetate 74:26); IR (ATR) ν_{max} 2914, 2853, 1737, 1242, 1205, 813, 785 cm^{-1}; ^{1}H NMR ($CDCl_3$, 400 MHz) δ 6.43 (2H, s, H-Ar), 2.90 (2H, m, H-3α), 2.51-2.43 (4H, m, H-4α and H-16β), 1.03 (6H, s, H-19), 0.88 (6H, s, H-18); ^{13}C NMR ($CDCl_3$, 100 MHz) δ 221.0 (2 x C), 149.1 (2 x C), 125.4 (2 x C), 113.1 (2 x CH), 78.4 (2 x C), 51.6 (2 x CH), 47.9 (2 x C), 43.8 (2 x CH), 42.9 (2 x C), 35.9 (2 x CH_2), 34.6 (2 x CH), 34.4 (2 x CH_2), 32.6 (2 x CH), 31.6 (2 x CH_2), 30.8 (2 x CH_2), 28.7 (2 x CH_2), 27.9 (2 x CH_2), 27.1 (2 x CH_2), 21.8 (2 x CH_2), 20.6 (2 x CH_2), 17.6 (2 x CH_3), 13.8 (2 x CH_3); HRMS *m/z* 651.4401 (calcd for $C_{44}H_{59}O_4^+$, 651.4408).

Compound 11c

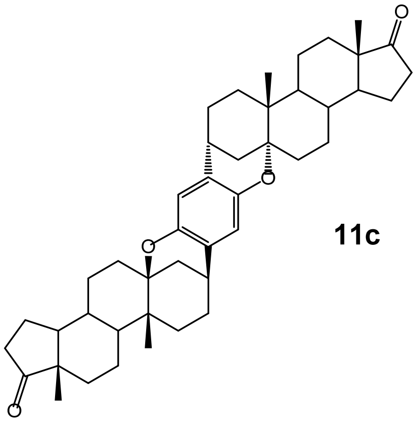

11c

White solid; Rf = 0.71 (3 x hexane/ethyl acetate 74:26); IR (ATR) ν_{max} 2929, 2870, 1737, 1241, 1194, 1152, 785 cm^{-1}; ^{1}H NMR ($CDCl_3$, 400 MHz) δ 6.43 (2H, s, H-Ar), 2.91 (1H, m, H-3α), 2.85 (1H, m, H-3β), 1.03 (3H, s, H-19), 1.01 (3H, s, H-19'), 0.88 (s, 6H, H-18 and H-18'); ^{13}C NMR ($CDCl_3$, 100 MHz) δ 221.4 (C), 220.9 (C), 149.0 (C), 148.7 (C), 126.1 (C), 125.2 (C), 113.0 (CH), 112.7 (CH), 78.3 (C), 78.1 (C), 51.6 (CH), 51.2 (CH), 47.8 (2 x C), 46.1 (CH), 43.9 (CH), 42.9 (C), 42.1 (C), 35.9 (2 x CH_2), 34.5 (CH), 34.4 (CH), 34.4 (CH_2), 33.6 (CH_2), 32.5 (2 x CH), 32.0 (CH_2), 31.6 (CH_2), 31.5 (CH_2), 30.7 (CH_2), 29.5 (CH_2), 29.4 (CH_2), 28.8 (CH_2), 27.9 (CH_2), 27.0 (CH_2), 24.8 (CH_2), 21.8 (CH_2), 21.7 (CH_2), 20.6 (CH_2), 20.0 (CH_2), 17.6 (CH_3), 16.2 (CH_3), 13.8 (2xCH_3); HRMS *m/z* 651.4403 (calcd for $C_{44}H_{59}O_4^+$, 651.4408).

Compound 12

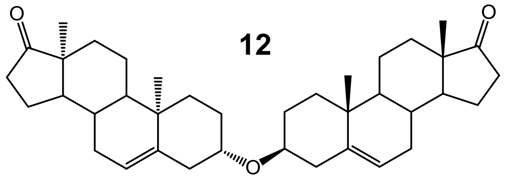

12

Colorless crystals (hexane/CH_2Cl_2); mp 268–269 °C; Rf = 0.45 (3 x benzene/ethyl acetate 94:6); IR (ATR) ν_{max} 2931, 2895, 1731, 1094, 1058, 1005cm^{-1}; ^{1}H NMR ($CDCl_3$, 400 MHz) δ 5.38 (2H, m, H-6), 3.30 (2H, m, H-3α), 2.47 (2H, dd, *J* = 19.2 Hz, *J* = 8.6 Hz, H-16β), 1.04 (6H, s, H-19), 0.89 (6H, s, H-18); ^{13}C NMR ($CDCl_3$, 100 MHz) δ 221.2 (2 x C), 141.5 (2 x C), 120.6 (2 x CH), 76.2 (2 x CH), 51.8 (2 x CH), 50.3 (2 x CH), 47.5 (2 x C), 40.0 (2 x CH_2), 37.3 (2 x CH_2), 37.0 (2 x C), 35.8 (2 x CH_2), 31.5 (2 x CH), 31.4 (2 x CH_2), 30.8 (2 x CH_2), 29.3 (2 x CH_2), 21.9 (2 x CH_2), 20.3 (2 x CH_2), 19.4 (2 x CH_3), 13.5 (2 x CH_3).

Diosgenin derived hydroquinone mono steroidal ether

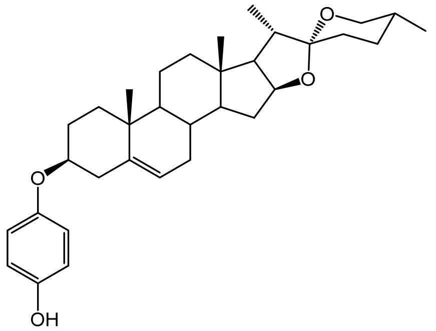

Colorless crystals (hexane/CH_2Cl_2); mp 165–166 °C; Rf = 0.35 (hexane/ethyl acetate 88:12); IR (ATR) ν_{max} 3309, 2929, 1507, 1210, 1046, 1012, 830 cm^{-1}; ^{1}H NMR ($CDCl_3$, 400 MHz) δ 6.80 (2H, d, *J* = 9.0 Hz, H-Ar), 6.75 (2H, d, *J* = 9.0 Hz, H-Ar), 5.38 (1H, m, H-6), 4.76 (1H, bs, -OH), 4.43 (1H, m, H-16), 3.97 (1H, m, H-3α), 3.49 (1H, m, H-26β), 3.39 (1H, t, *J* = 10.9 Hz, H-26α), 1.07 (3H, s, H-19), 0.99 (3H, d, *J* = 6.9 Hz, H-21), 0.81 (3H, s, H-18), 0.80 (3H, d,

J = 6.4 Hz, H-27); ^{13}C NMR ($CDCl_3$, 100 MHz) δ 151.6 (C), 149.8 (C), 140.5 (C), 121.9 (CH), 117.8 (CH), 116.0 (CH), 109.4 (C), 80.8 (CH), 78.3 (CH), 66.9 (CH_2), 62.1 (CH), 56.5 (CH), 50.1 (CH), 41.6 (CH), 40.3 (C), 39.8 (CH_2), 38.8 (CH_2), 37.1 (CH_2), 37.0 (C), 32.1 (CH_2), 31.8 (CH_2), 31.42 (CH), 31.37 (CH_2), 30.3 (CH), 28.8 (CH_2), 28.3 (CH_2), 20.9 (CH_2), 19.4 (CH_3), 17.1 (CH_3), 16.3 (CH_3), 14.5 (CH_3); HRMS *m/z* 507.3476 (calcd for $C_{33}H_{47}O_4^+$, 507.3469).

4. Assignments of ^{1}H and ^{13}C NMR signals for compounds 11a and 11b

Table S1. ^{1}H NMR and ^{13}C NMR data for dimers **11a**, **11b**, and 5α- and 5β-androstanones [8] measured in $CDCl_3$

	11a		5α-androstan-17-one		5β-androstan-17-one		11b	
No	^{1}H	^{13}C	^{1}H	^{13}C	^{1}H	^{13}C	^{1}H	^{13}C
1	1.26, 1.20	29.5 (CH_2)	1.67, 0.89	38.7 (CH_2)	1.75, 0.91	37.6 (CH_2)	1.47, 1.22	27.9 (CH_2)
2	1.94, 1.60	29.3 (CH_2)	1.50, 1.41	22.1 (CH_2)	1.37, 1.27	21.3 (CH_2)	1.76, 1.48	28.7 (CH_2)
3	2.84	32.6 (CH)	1.65, 1.22	26.7 (CH_2)	1.72, 1.21	27.0 (CH_2)	2.90	32.6 (CH)
4	2.08, 1.40	32.2 (CH_2)	1.29, 1.29	28.7 (CH_2)	1.72, 1.22	27.1 (CH_2)	2.48, 1.43	30.8 (CH_2)
5	-	78.1 (C)	1.07	47.1 (CH)	1.31	43.7 (CH)	-	78.4 (C)
6	1.74, 1.45	33.7 (CH_2)	1.25, 1.25	29.0 (CH_2)	1.90, 1.27	27.2 (CH_2)	1.78, 1.21	27.1 (CH_2)
7	1.74, 1.58	24.8 (CH_2)	1.78, 0.97	31.0 (CH_2)	1.52, 1.18	25.5 (CH_2)	2.07, 1.48	34.4 (CH_2)
8	1.63	34.4 (CH)	1.55	35.1 (CH)	1.58	35.5 (CH)	1.66	34.6 (CH)
9	1.63	46.1 (CH)	0.72	54.9 (CH)	1.47	40.9 (CH)	1.53	43.8 (CH)
10	-	42.1 (C)	-	36.4 (C)	-	35.6 (C)	-	42.9 (C)
11	1.52, 1.29	20.0 (CH_2)	1.67, 1.27	20.1 (CH_2)	1.55, 1.26	20.1 (CH_2)	1.70, 1.30	20.6 (CH_2)
12	1.80, 1.29	31,6 (CH_2)	1.79, 1.23	31,6 (CH_2)	1.80, 1.27	31,8 (CH_2)	1.86, 1.32	31,6 (CH_2)

13	-	47.9 (C)	-	47.8 (C)	-	47.9 (C)	-	47.9 (C)
14	1.46	51.2 (CH)	1.27	51.6 (CH)	1.36	51.6 (CH)	1.38	51.6 (CH)
15	1.98, 1.50	21.8 (CH_2)	1.91, 1.60	21.8 (CH_2)	1.93, 1.49	21.8 (CH_2)	1.95, 1.55	21.8 (CH_2)
16	2.46, 2,10	35.9 (CH_2)	2.45, 2,03	35.9 (CH_2)	2.43, 2,06	36.0 (CH_2)	2.49, 2,13	35.9 (CH_2)
17	-	221.5 (C)	-	221.5 (C)	-	221.6 (C)	-	221.0 (C)
18	0.88	13.8 (CH_3)	0.86	13.8 (CH_3)	0.85	13.8 (CH_3)	0.88	13.8 (CH_3)
19	1.04	16.1 (CH_3)	0.81	12.2 (CH_3)	0.95	24.2 (CH_3)	1.03	17.6 (CH_3)
Ar_{CH}	6.41	112.8 (CH)					6.43	113.2 (CH)
Ar_C	-	148.4 (C)					-	149.1 (C)
Ar_C	-	126.1 (C)					-	125.4 (C)

5. References

[1] N. Dhingra, T. R. Bhardwaj, N. Mehta, T. Mukhopadhyay, A. Kumar, M. Kumar, *Arch Pharm Res* **2011**, *34,* 7, 1055-1063.
[2] W. Shuping, J. Zhiqin, L. Heting, Y. Li, Z. Daixun, *Molecules* **2001**, *6*, 52-60.
[3] P. J. Wallis, W. P. Gates, A. F. Patti, J. L. Scott, E. Teoh, *Green Chemistry* **2007**, *9*, 980-986.
[4] K. Ebitani, T. Kawabata, K. Nagashima, T. Mizugaki, K. Kaneda, *Green Chemistry* **2000**, *2*, 157-160.
[5] T. Joseph, G. V. Shanbhag, S. B. Halligudi, *Journal of Molecular Catalysis A: Chemical* **2005**, *236*, 139-144.
[6] A. Zmysłowski, J. Sitkowski, K. Bus, K. Ofiara, A. Szterk, *Food Chemistry* **2020**, *329*, 127132.
[7] A. M. Tomkiel, J. Kowalski, J. Płoszyńska, L. Siergiejczyk, Z. Łotowski, A. Sobkowiak, J. W. Morzycki, *Steroids* **2014**, *82*, 60-67.
[8] Y. Yang, T. Haino, S. Usui, Y. Fukazawa, *Tetrahedron*, **1996**, *52*, 7, 2325–2336.

6. ^{1}H and ^{13}C NMR spectra of all new compounds

Compound 1

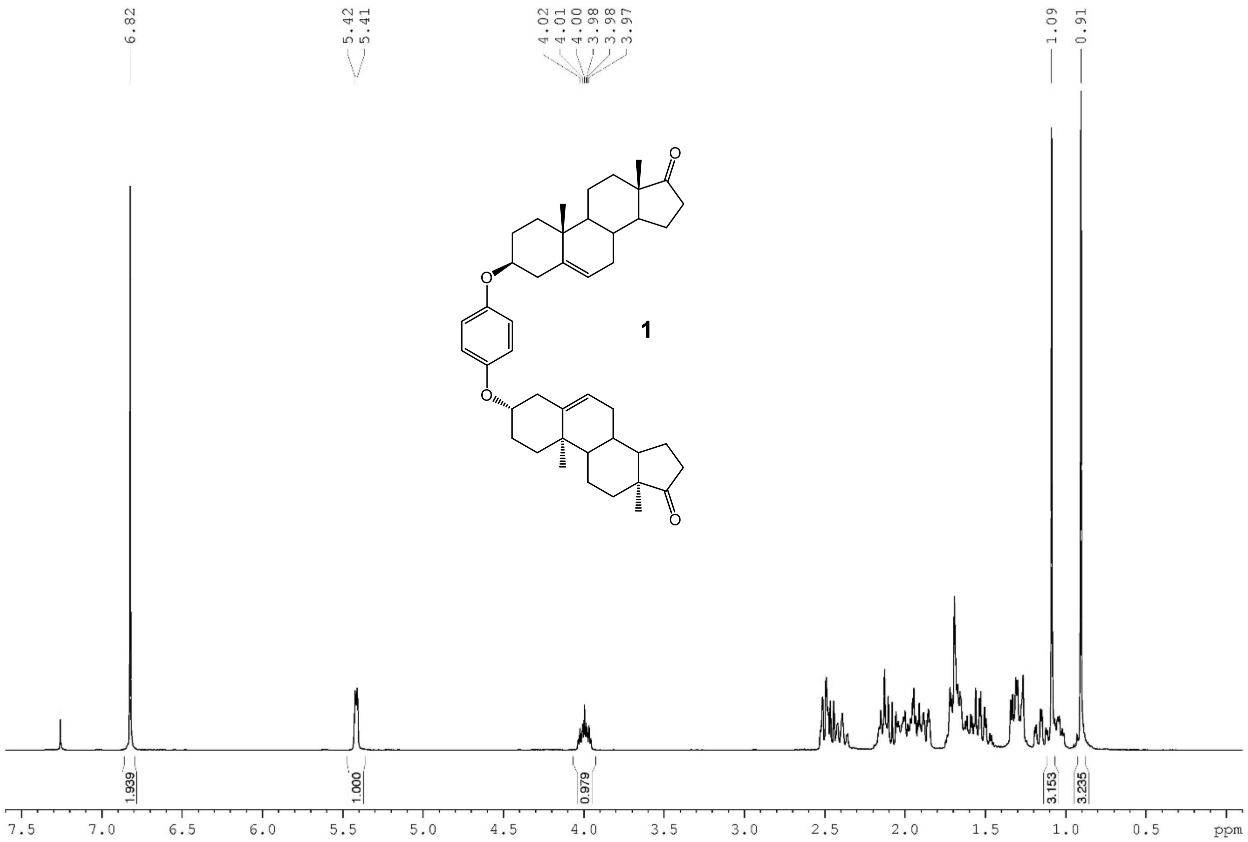

6.82
5.42
5.41
4.02
4.01
4.00
3.98
3.98
3.97
1.09
0.91
1
1.939
1.000
0.979
3.153
3.235
ppm

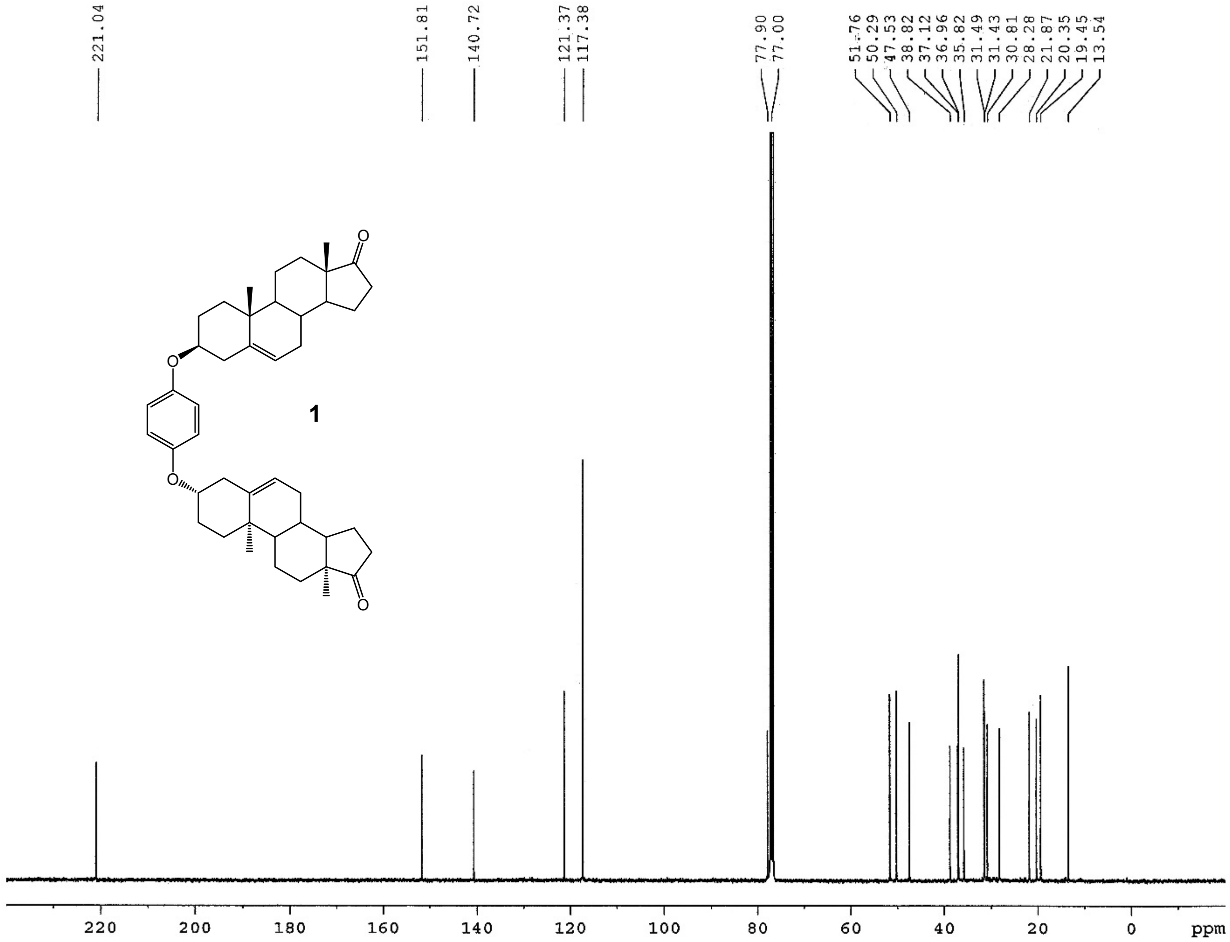

221.04
151.81
140.72
121.37
117.38
77.90
77.00
51.76
50.29
47.53
38.82
37.12
36.96
35.82
31.49
31.43
30.81
28.28
21.87
20.35
19.45
13.54
1
ppm

Compound 2

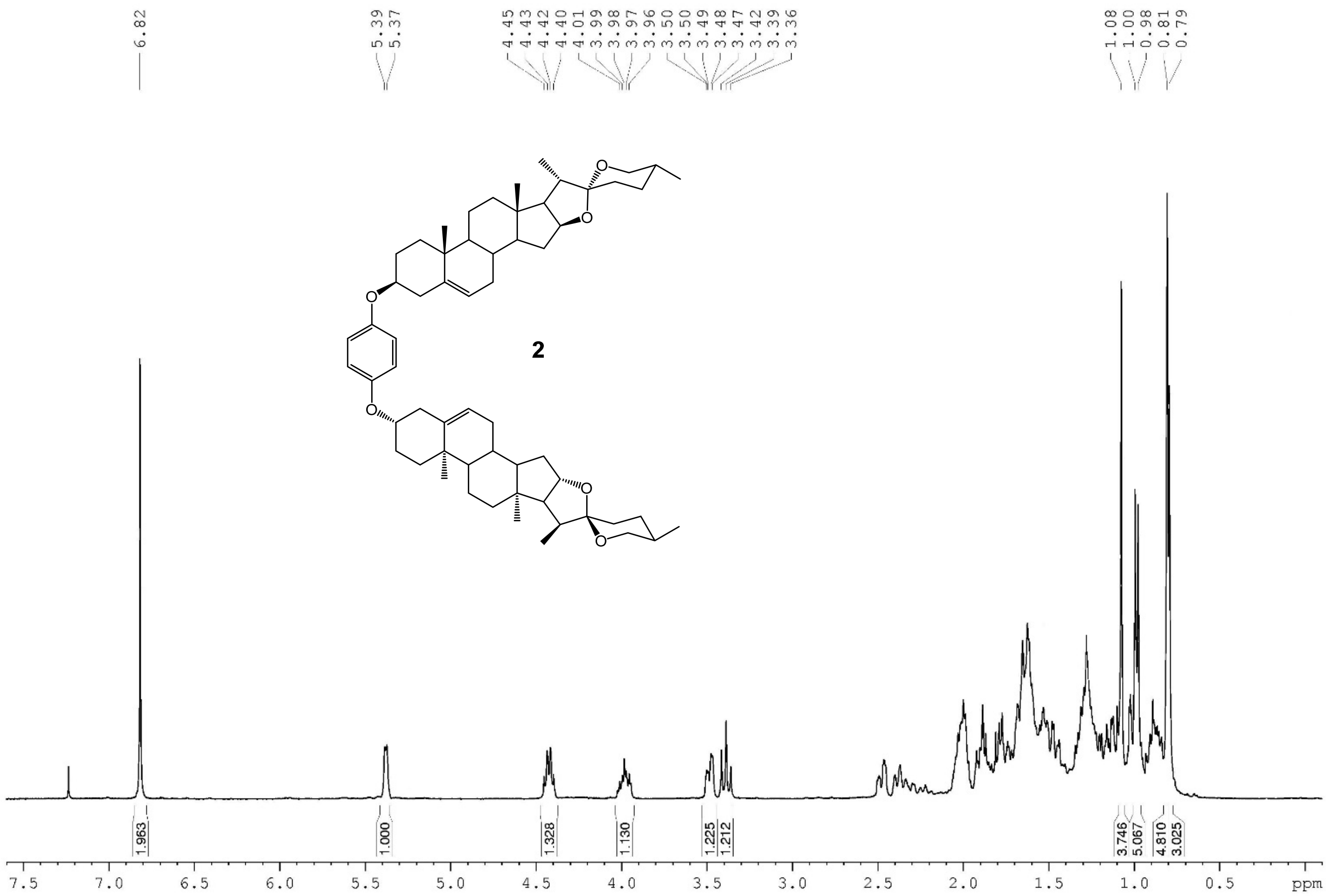
6.82
5.39
5.37
4.45
4.43
4.42
4.40
4.01
3.99
3.98
3.97
3.96
3.50
3.50
3.49
3.48
3.47
3.42
3.39
3.36
1.08
1.00
0.98
0.81
0.79
2
1.963
1.000
1.328
1.130
1.225
1.212
3.746
5.067
4.810
3.025
7.5
7.0
6.5
6.0
5.5
5.0
4.5
4.0
3.5
3.0
2.5
2.0
1.5
1.0
0.5
ppm

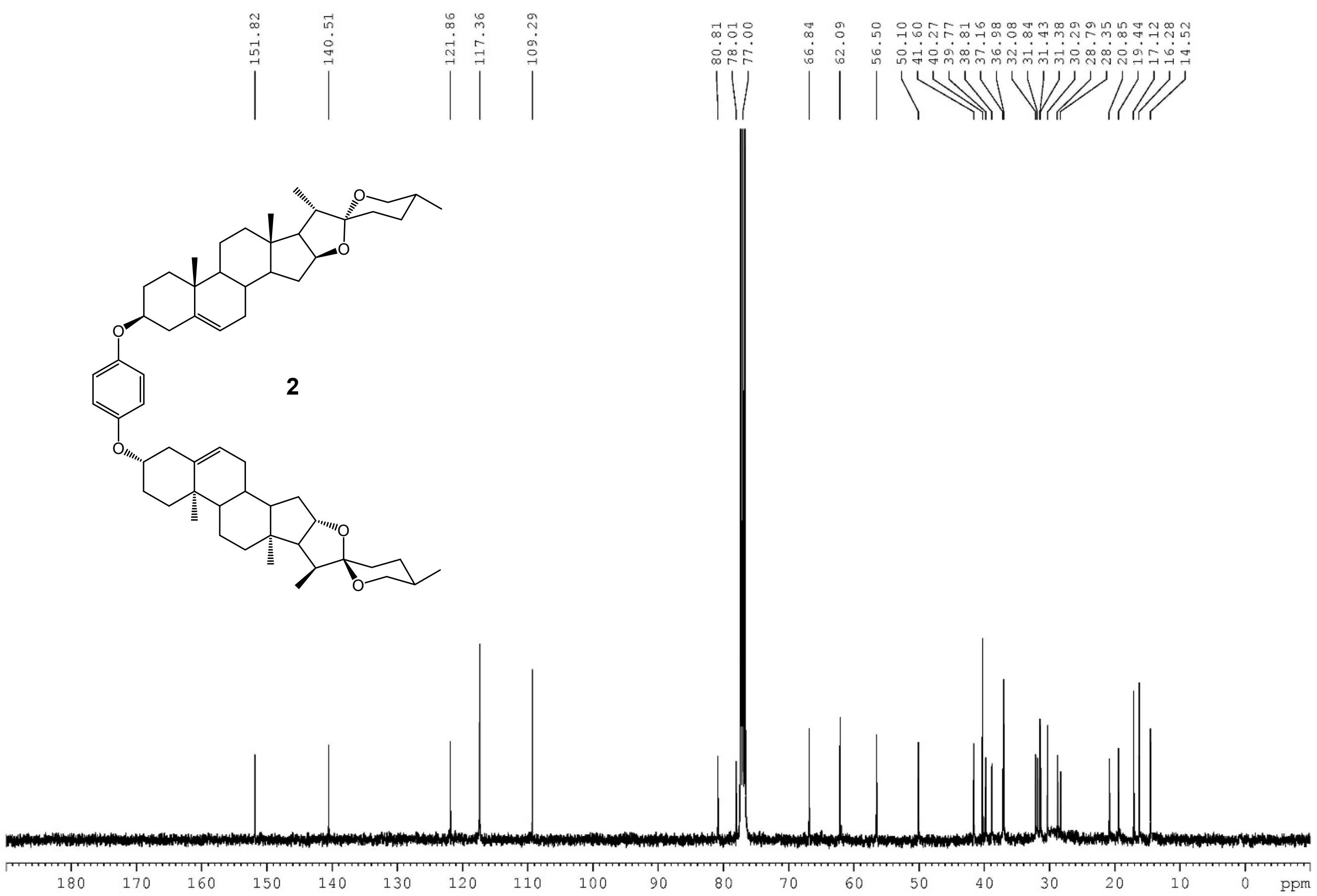
151.82
140.51
121.86
117.36
109.29
80.81
78.01
77.00
66.84
62.09
56.50
50.10
41.60
40.27
39.77
38.81
37.16
36.98
32.08
31.84
31.43
31.38
30.29
28.79
28.35
20.85
19.44
17.12
16.28
14.52
2
180
170
160
150
140
130
120
110
100
90
80
70
60
50
40
30
20
10
0
ppm

Compound 3

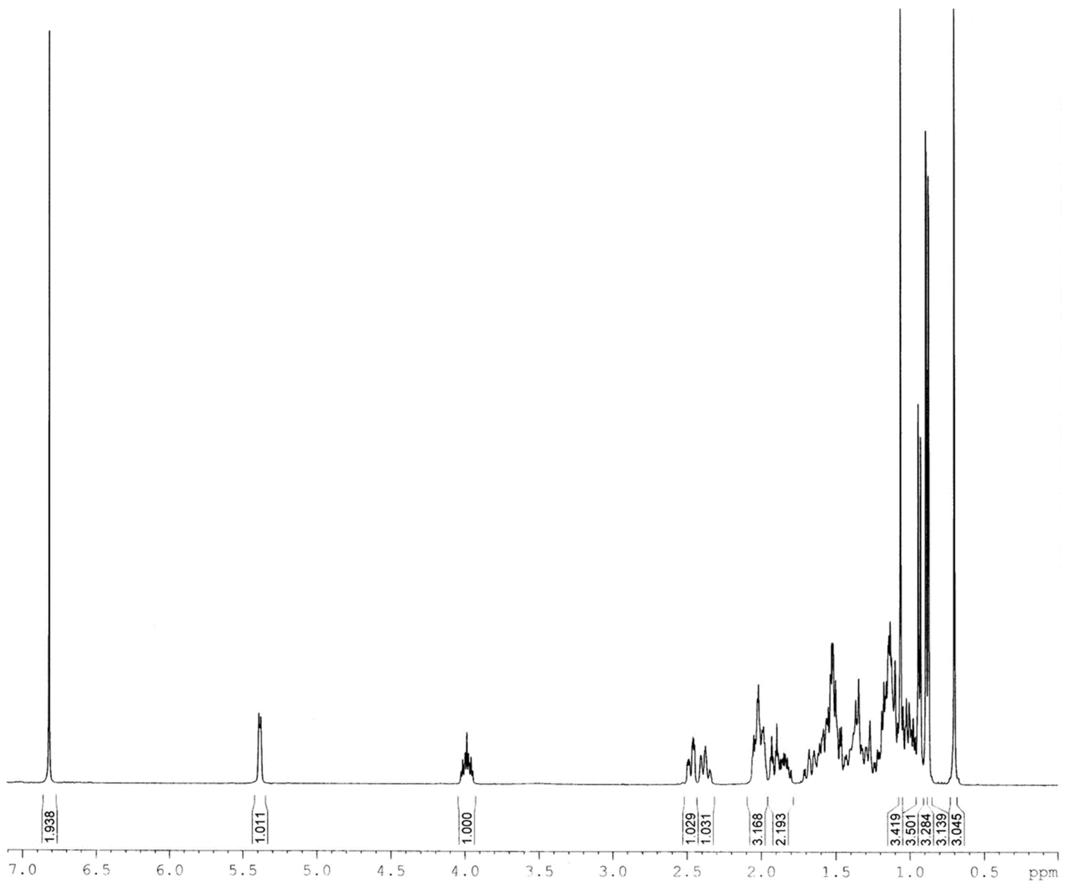
1.938
1.011
1.000
1.029
1.031
3.168
2.193
3.419
3.501
3.284
3.139
3.045
7.0 6.5 6.0 5.5 5.0 4.5 4.0 3.5 3.0 2.5 2.0 1.5 1.0 0.5 ppm

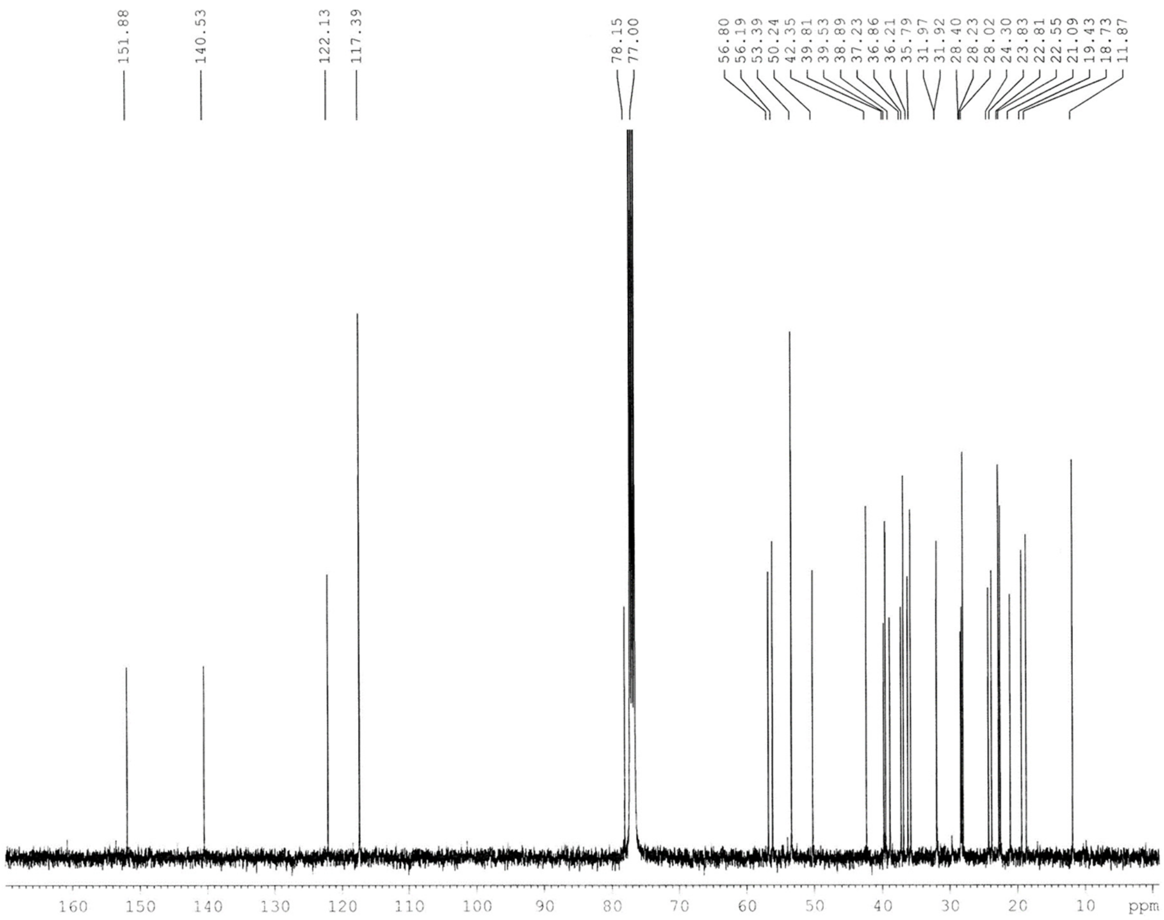
151.88
140.53
122.13
117.39
78.15
77.00
56.80
56.19
53.39
50.24
42.35
39.81
39.53
38.89
37.23
36.86
36.21
35.79
31.97
31.92
28.40
28.23
28.02
24.30
23.83
22.81
22.55
21.09
19.43
18.73
11.87
160 150 140 130 120 110 100 90 80 70 60 50 40 30 20 10 ppm

Compound 7

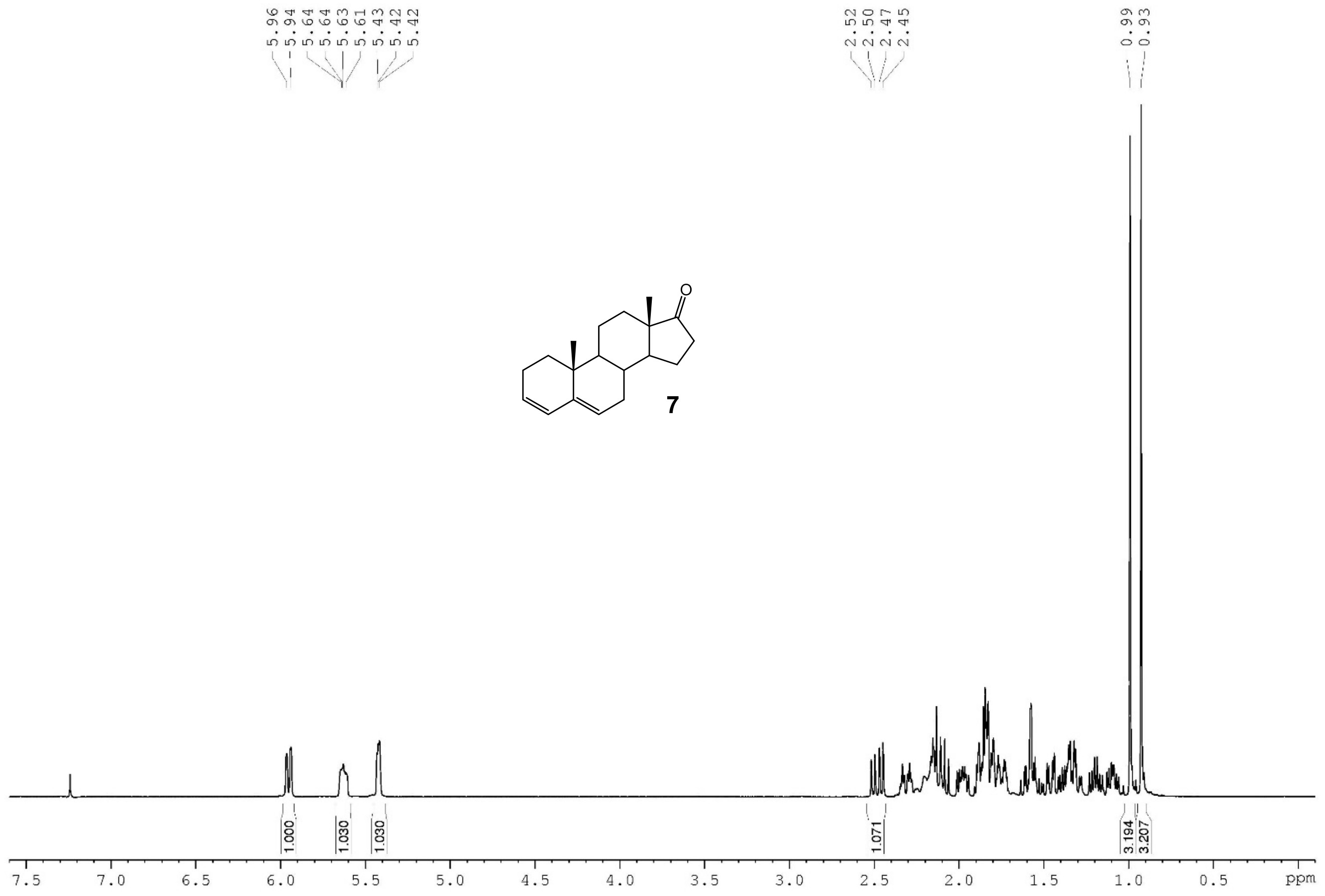
5.96
5.94
5.64
5.63
5.61
5.43
5.42
5.42
2.52
2.50
2.47
2.45
0.99
0.93
7
1.000
1.030
1.030
1.071
3.194
3.207
7.5 7.0 6.5 6.0 5.5 5.0 4.5 4.0 3.5 3.0 2.5 2.0 1.5 1.0 0.5 ppm

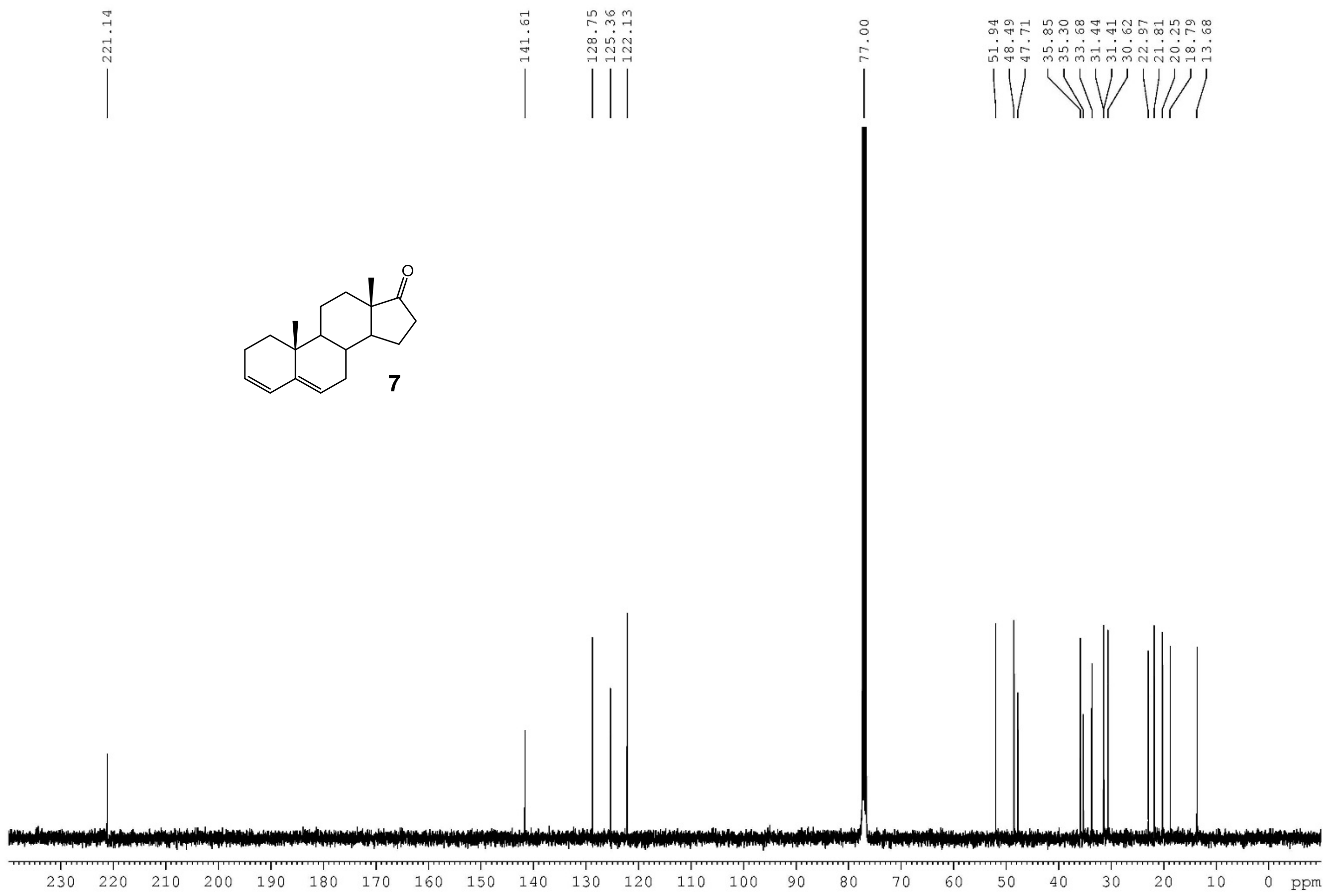
221.14
141.61
128.75
125.36
122.13
77.00
51.94
48.49
47.71
35.85
35.30
33.68
31.44
31.41
30.62
22.97
21.81
20.25
18.79
13.68
7
230 220 210 200 190 180 170 160 150 140 130 120 110 100 90 80 70 60 50 40 30 20 10 0 ppm

Compound 8

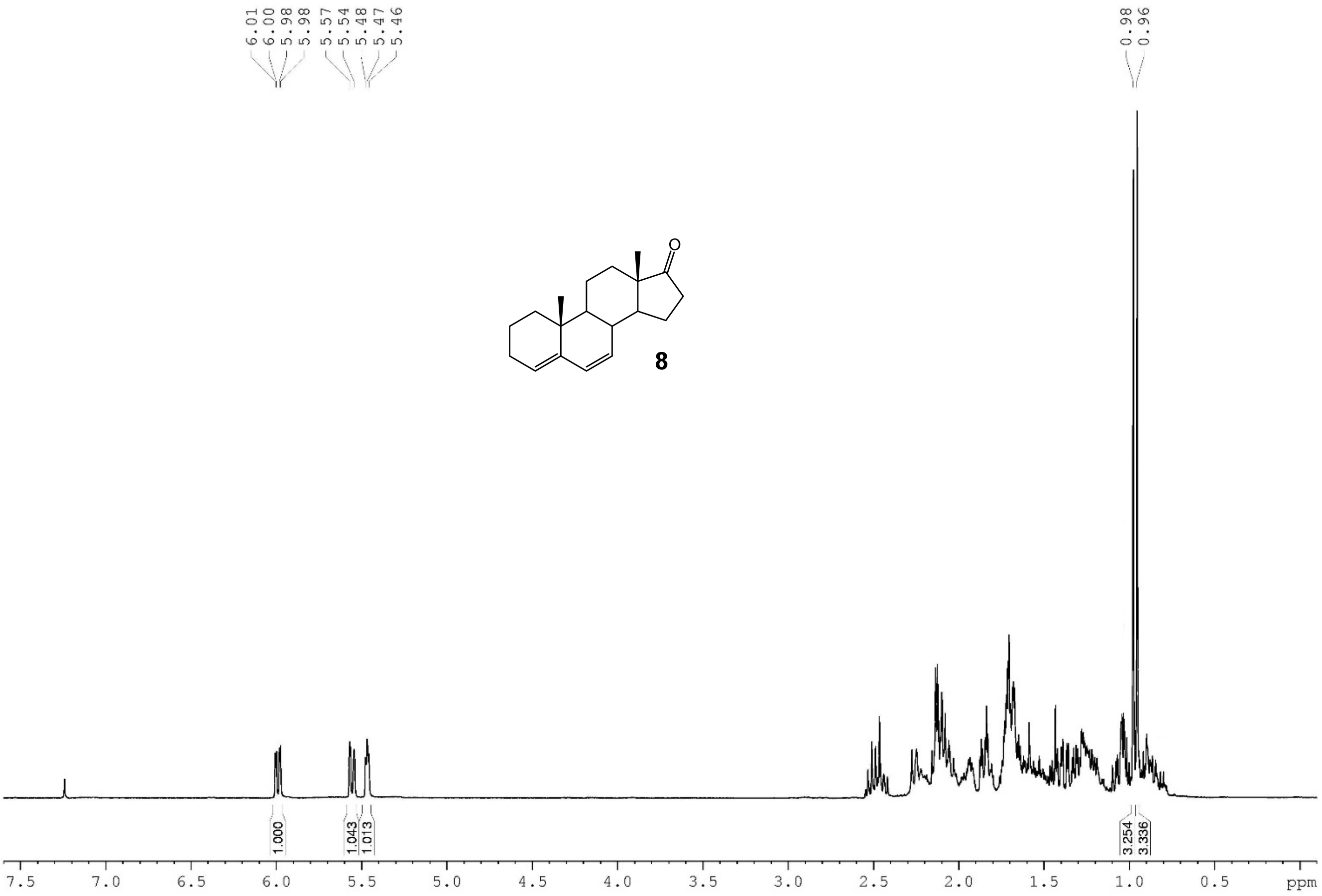
6.01 6.00 5.98 5.98 5.57 5.54 5.48 5.47 5.46
0.98 0.96
8
1.000 1.043 1.013 3.254 3.336
ppm

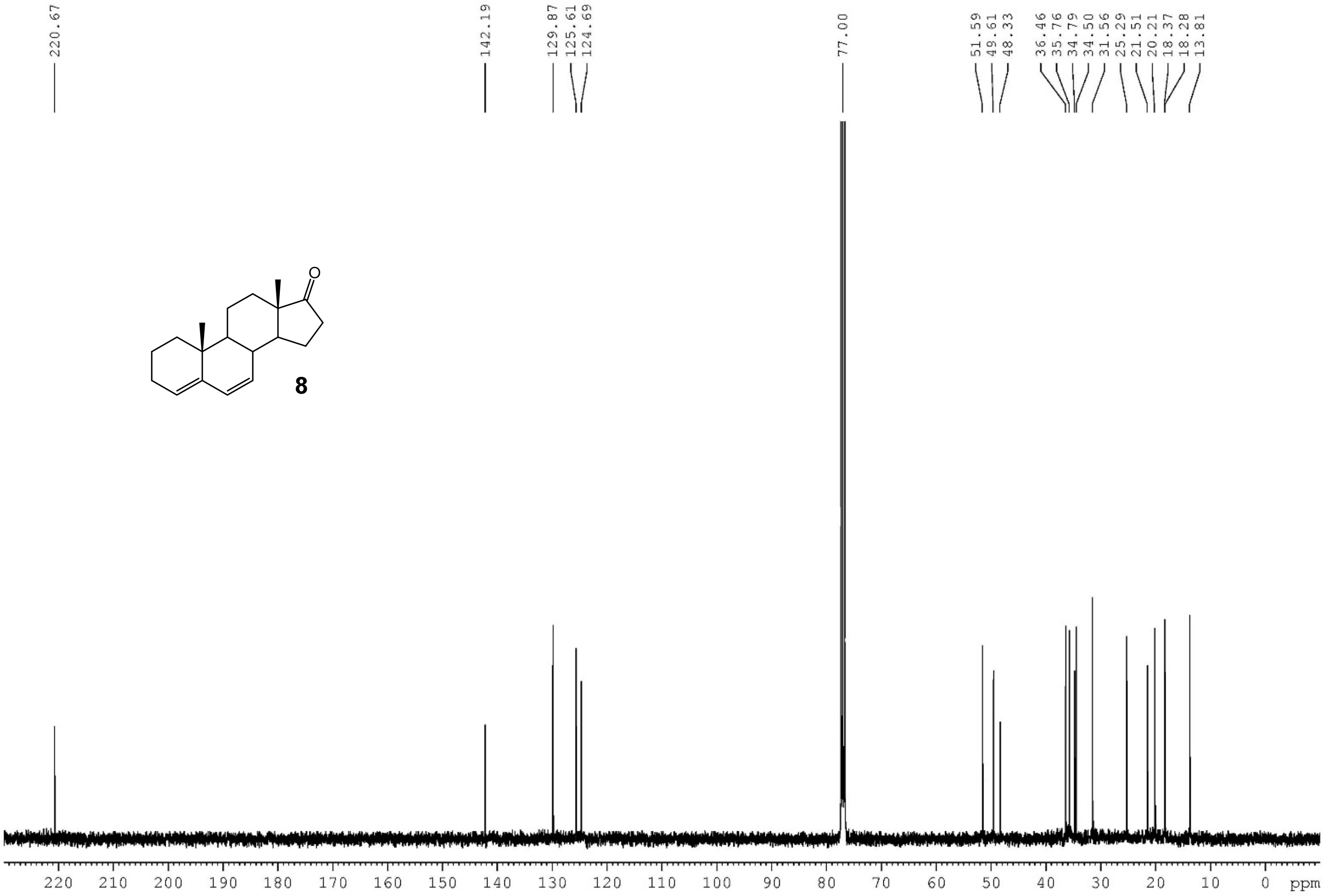
220.67
142.19
129.87 125.61 124.69
77.00
51.59 49.61 48.33
36.46 35.76 34.79 34.50 31.56 25.29 21.51 20.21 18.37 18.28 13.81
8
ppm

Compound 9

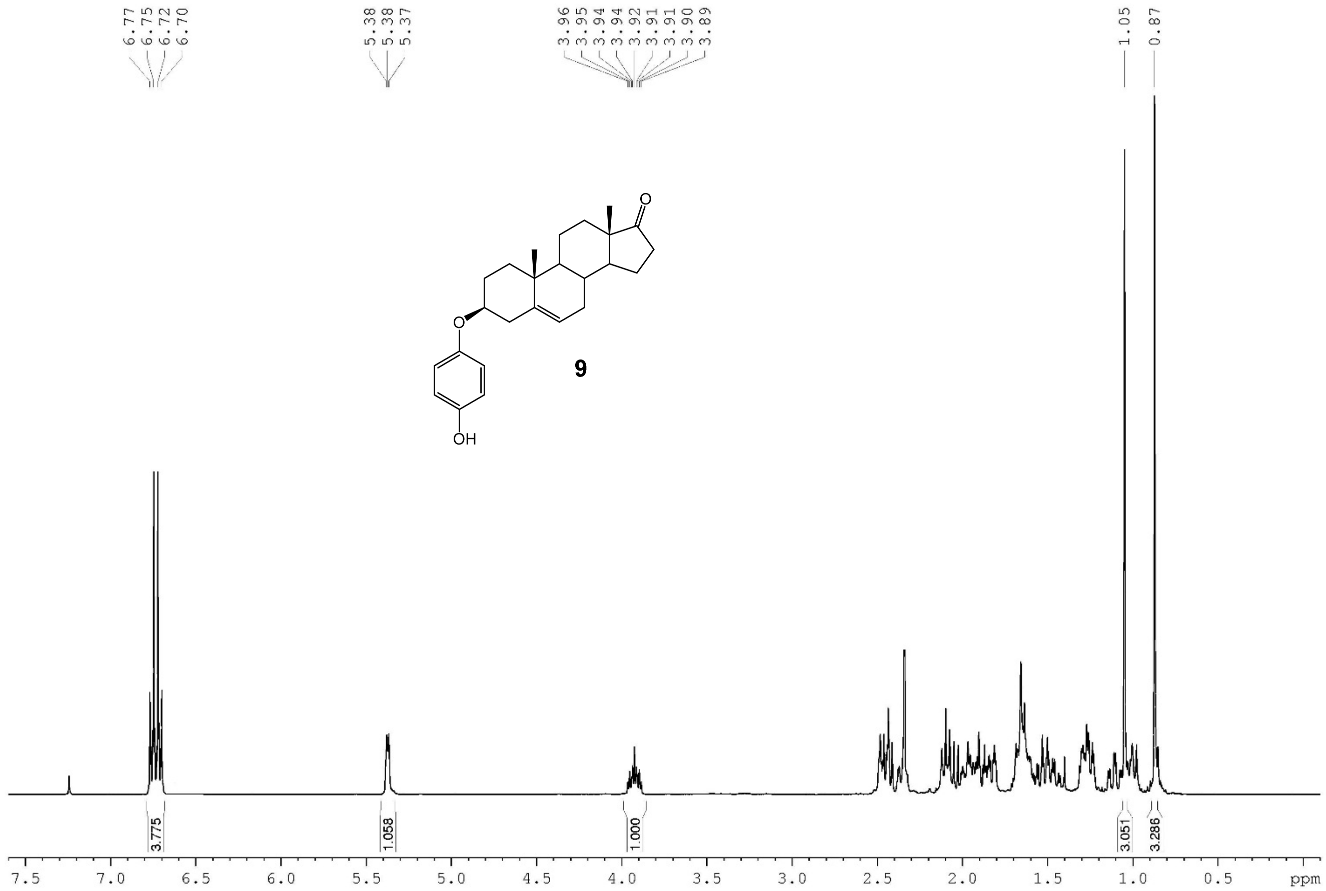

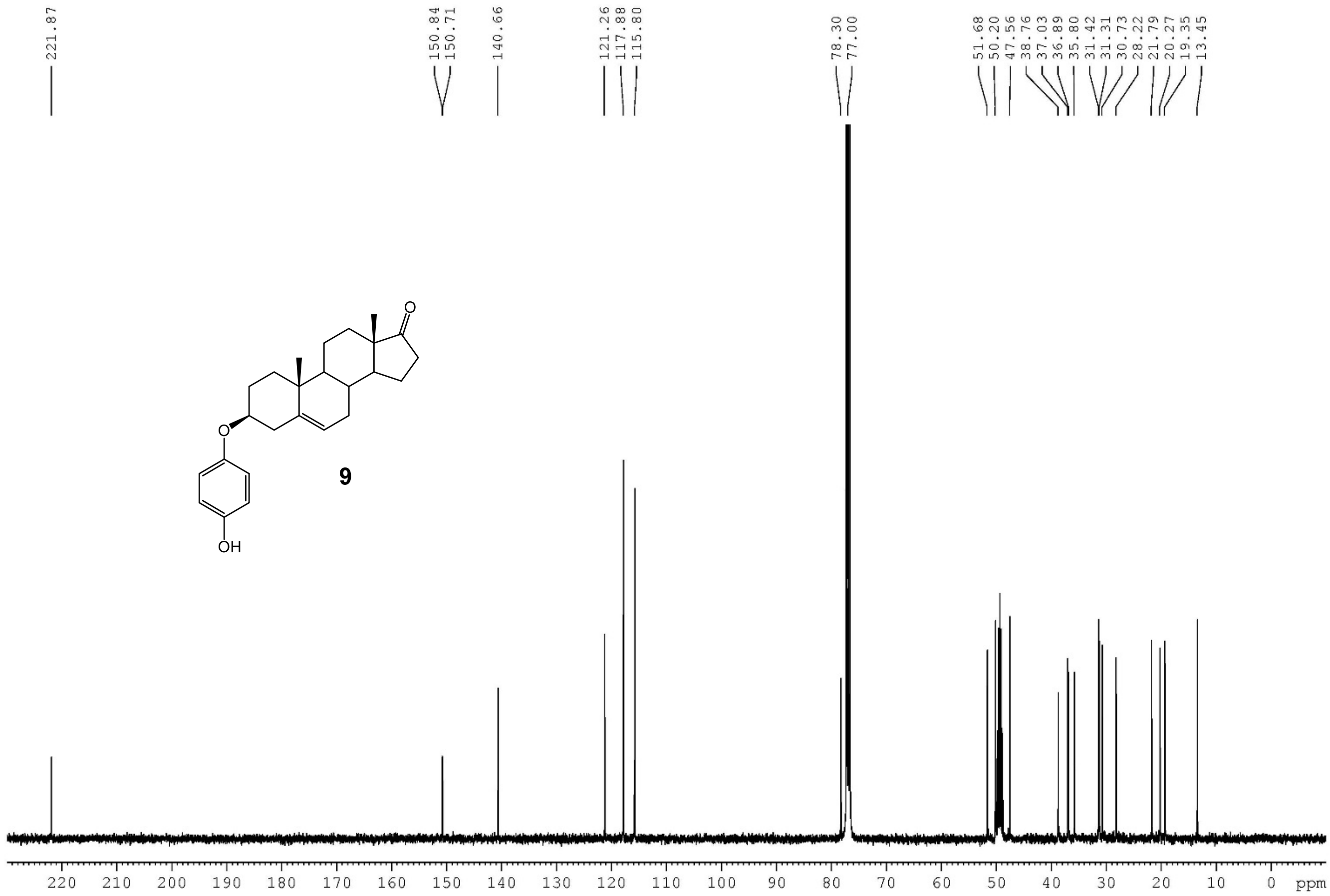

Compound 10a

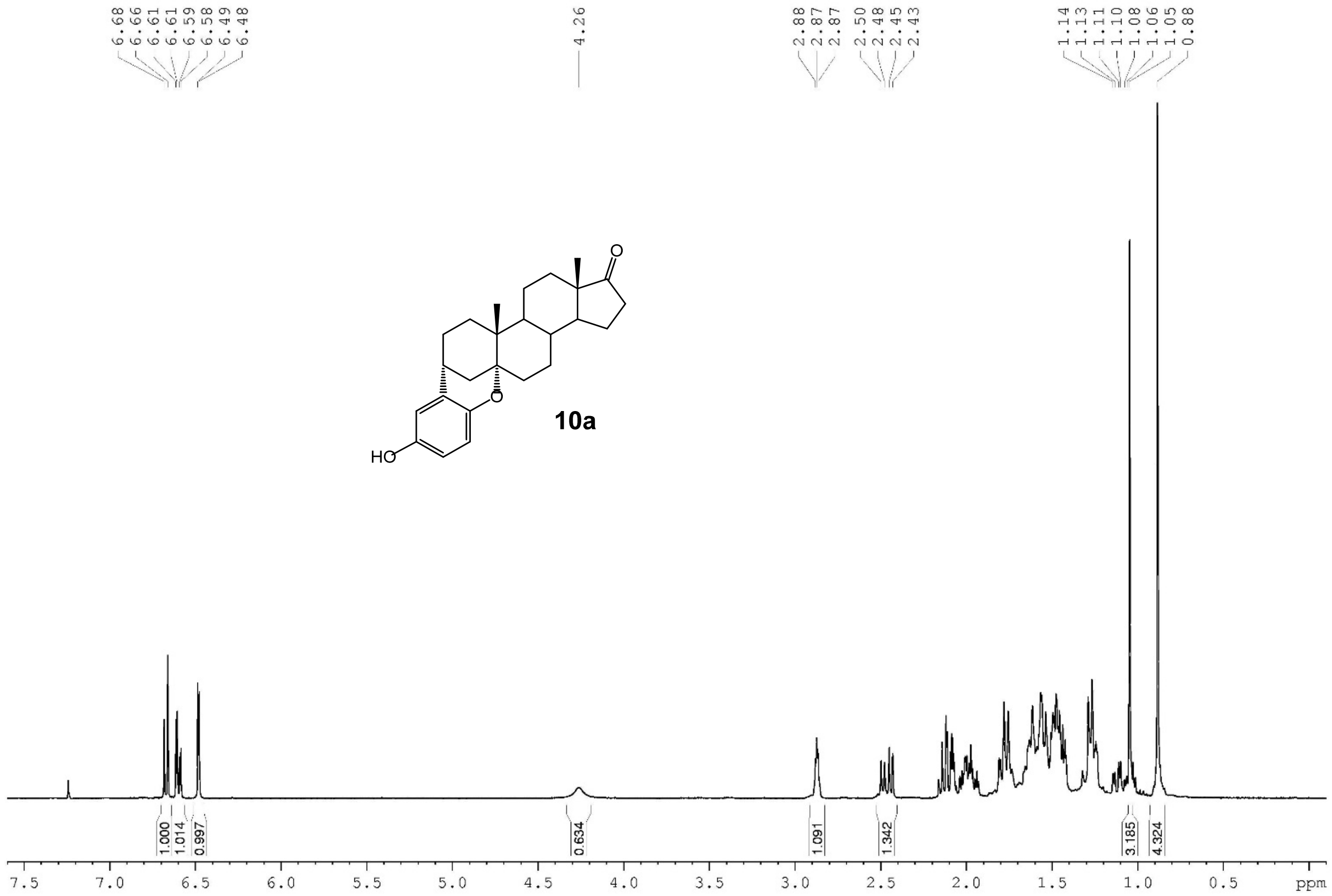

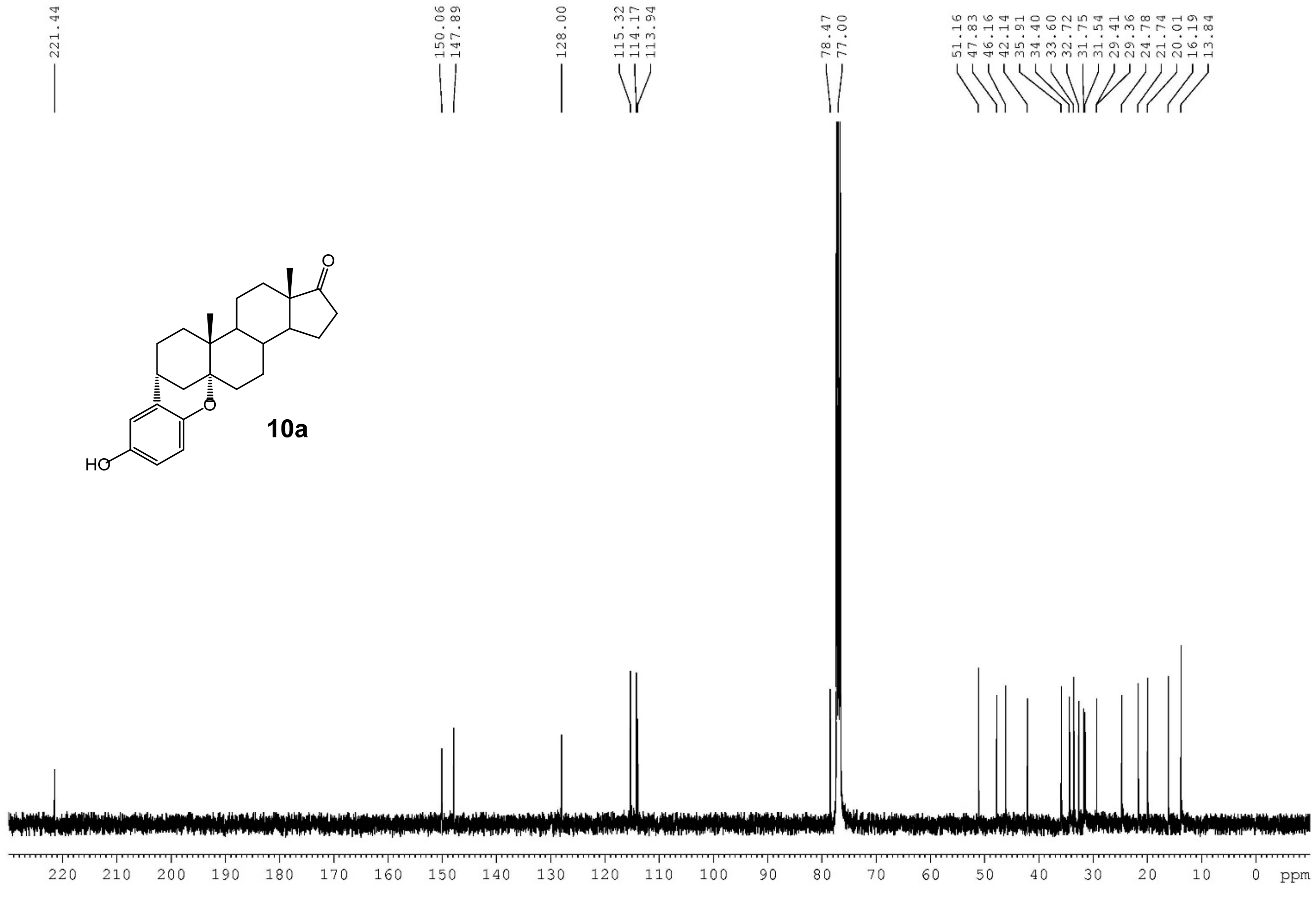

Compound 11a

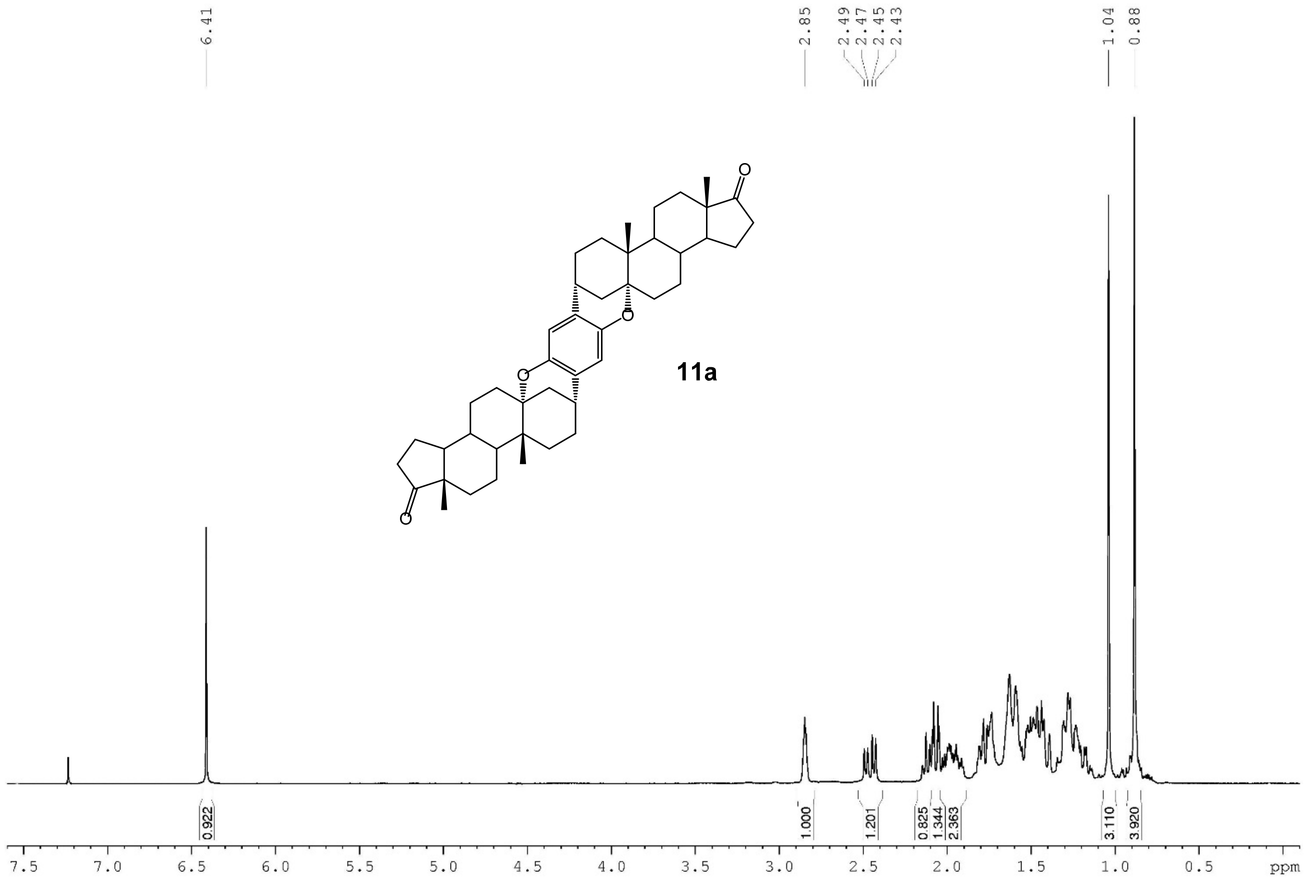
6.41
2.85
2.49
2.47
2.45
2.43
1.04
0.88
11a
0.922
1.000
1.201
0.825
1.344
2.363
3.110
3.920
7.5 7.0 6.5 6.0 5.5 5.0 4.5 4.0 3.5 3.0 2.5 2.0 1.5 1.0 0.5 ppm

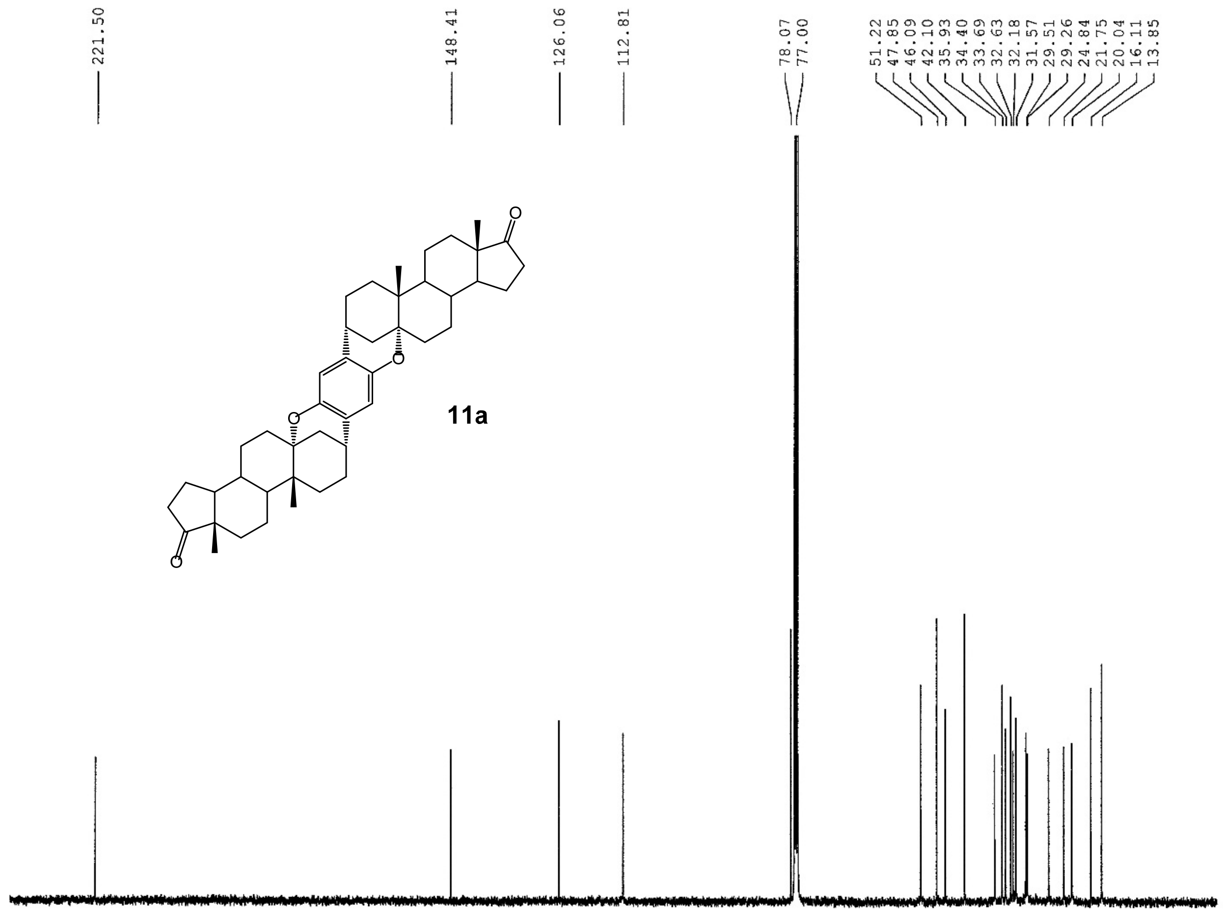
221.50
148.41
126.06
112.81
78.07
77.00
51.22
47.85
46.09
42.10
35.93
34.40
33.69
32.63
32.18
31.57
29.51
29.26
24.84
21.75
20.04
16.11
13.85
11a
220 200 180 160 140 120 100 80 60 40 20 0 ppm

^{1}H COSY NMR

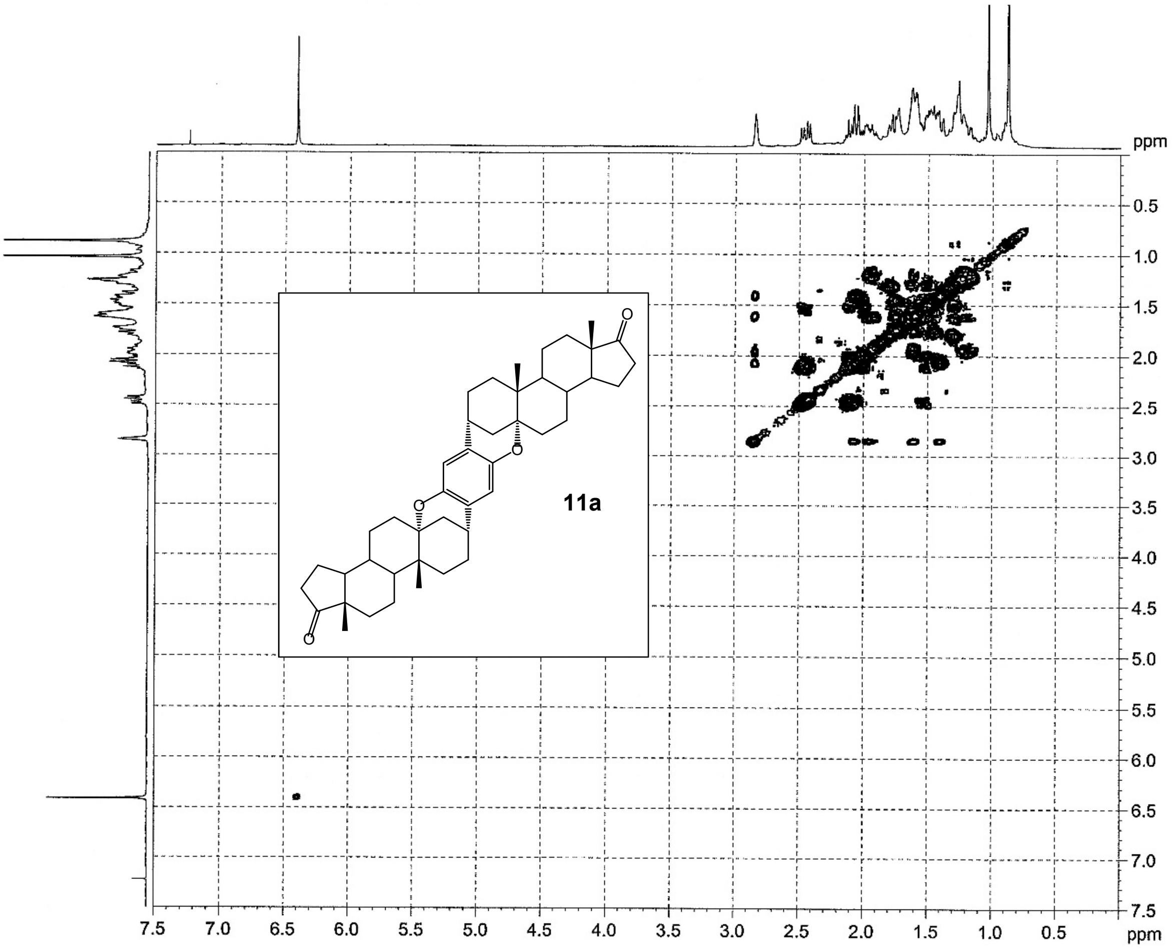

^{1}H-^{13}C HSQC NMR

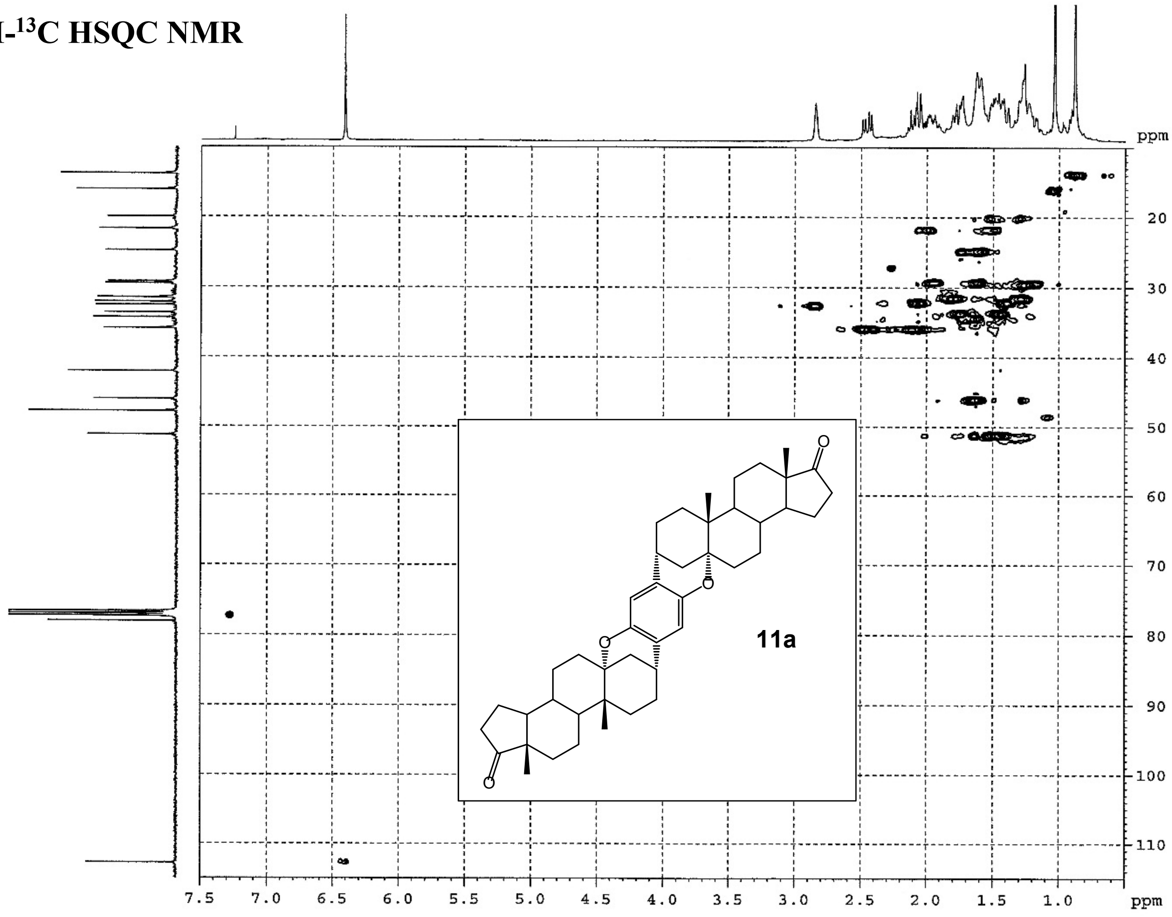

^{1}H-^{13}C HMBC NMR

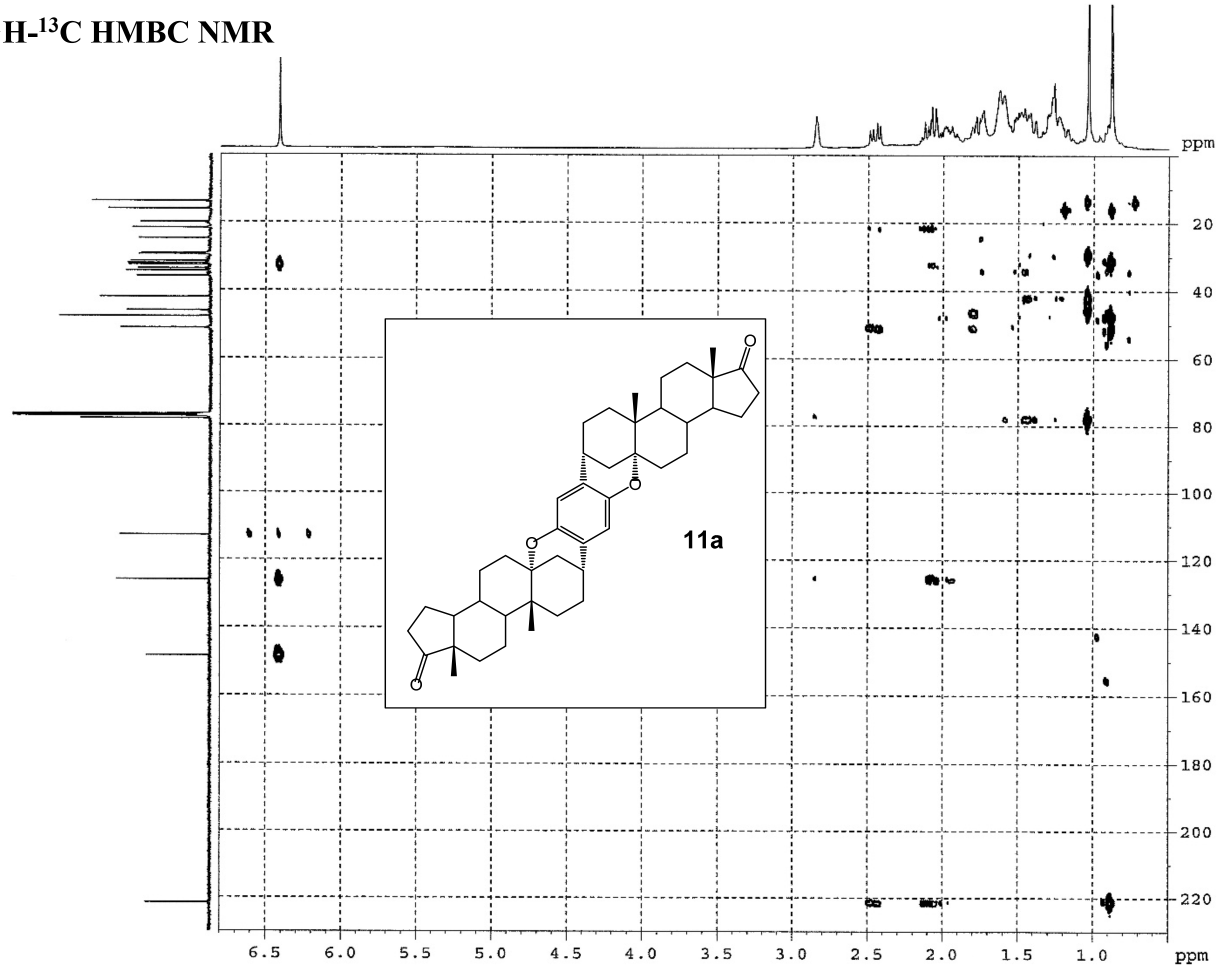

^{1}H-^{13}C HMQC NMR

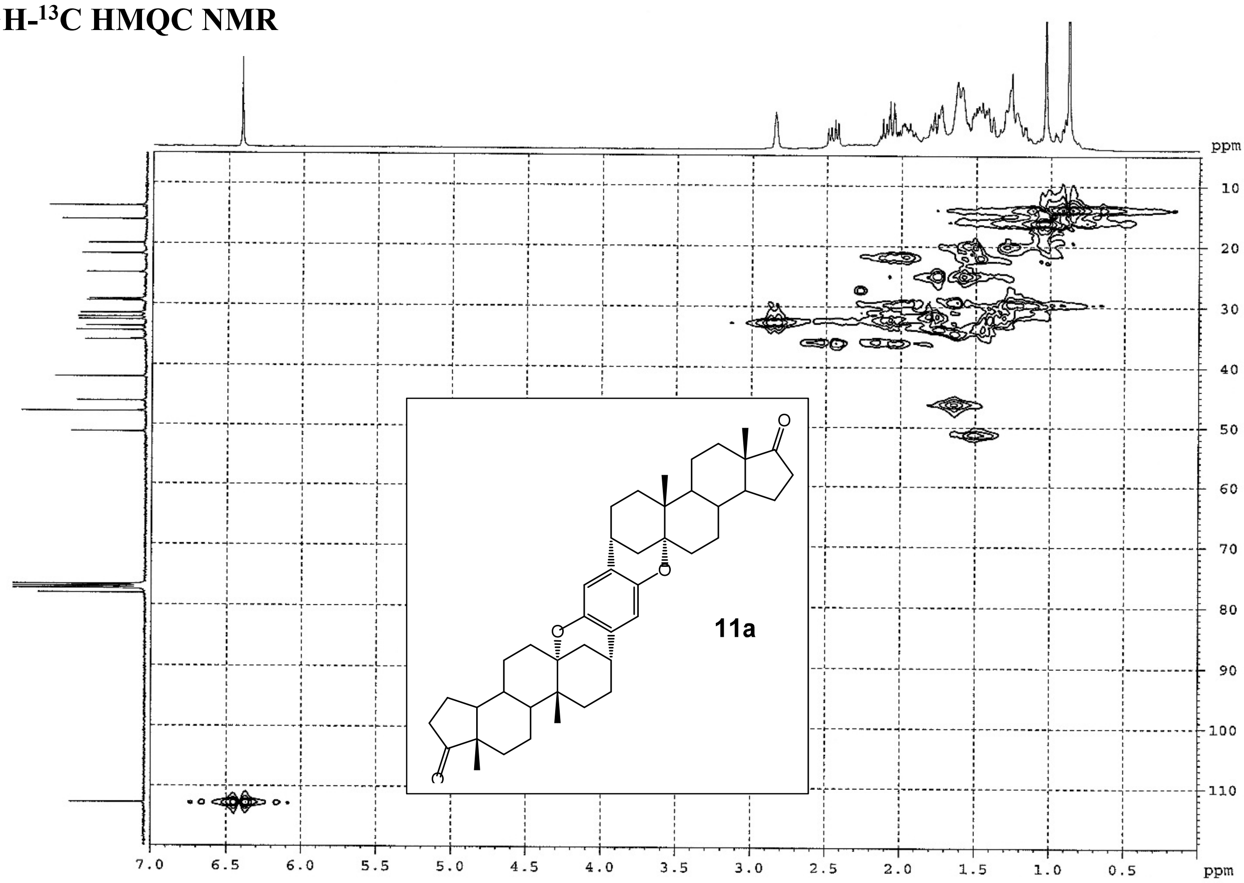

Compound 11b

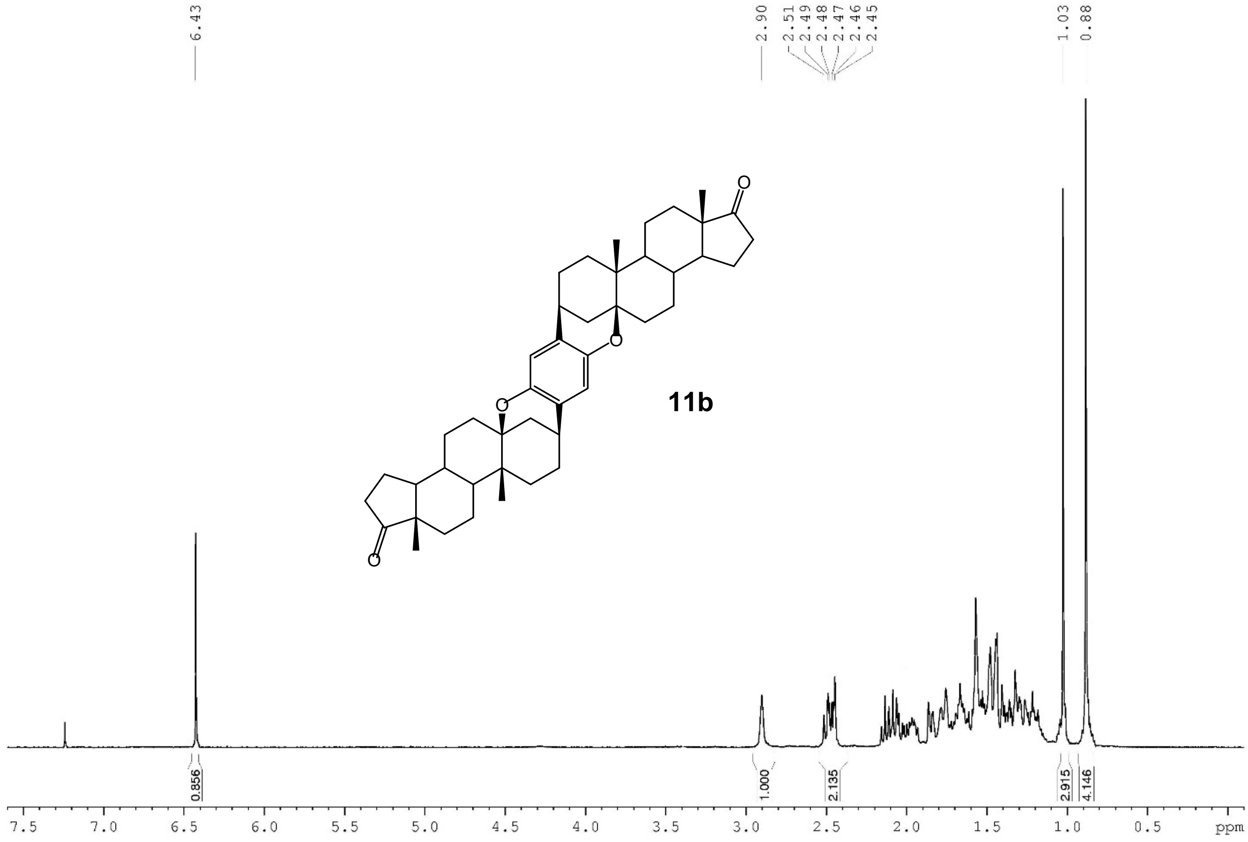

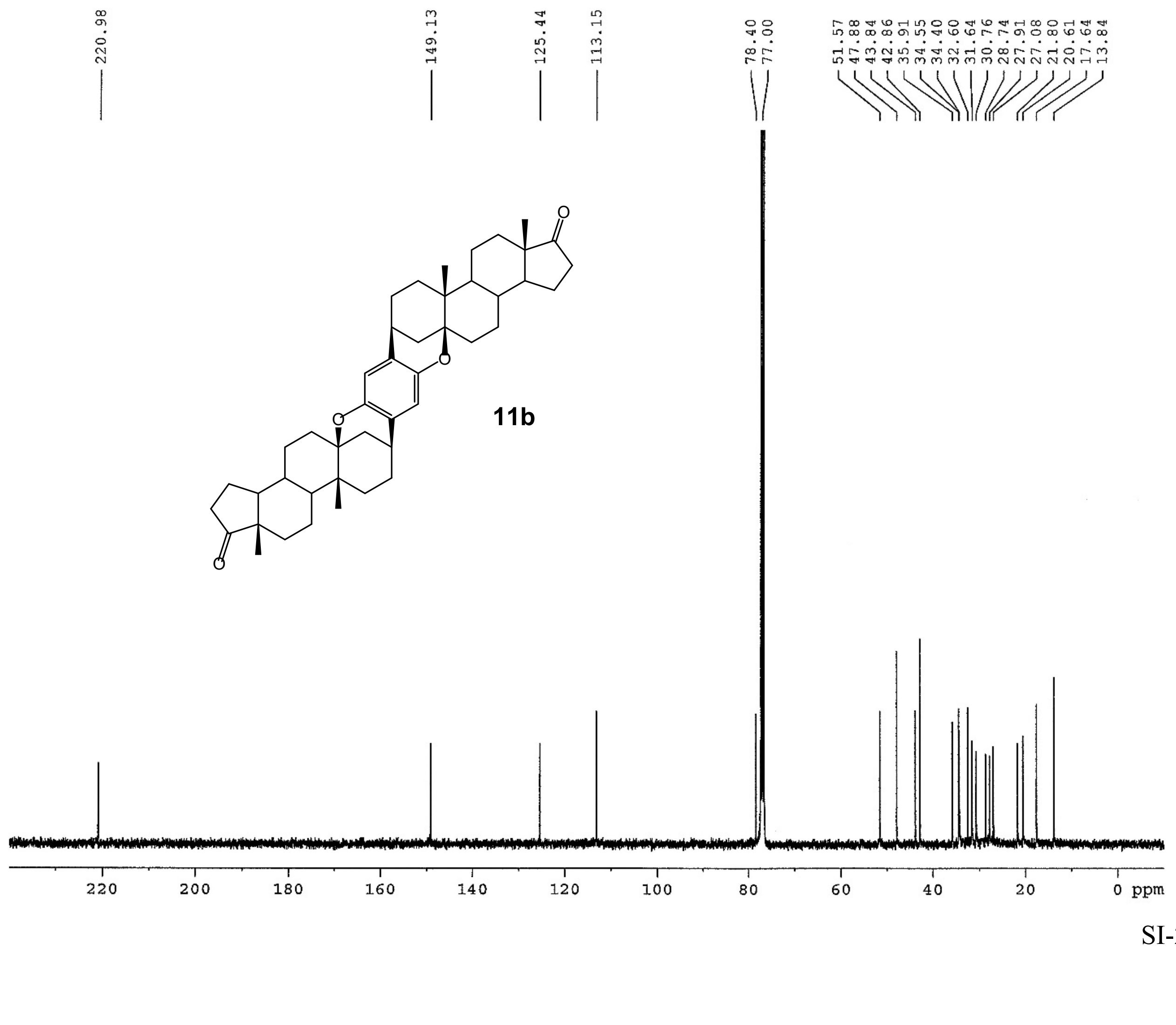

^{1}H COSY NMR

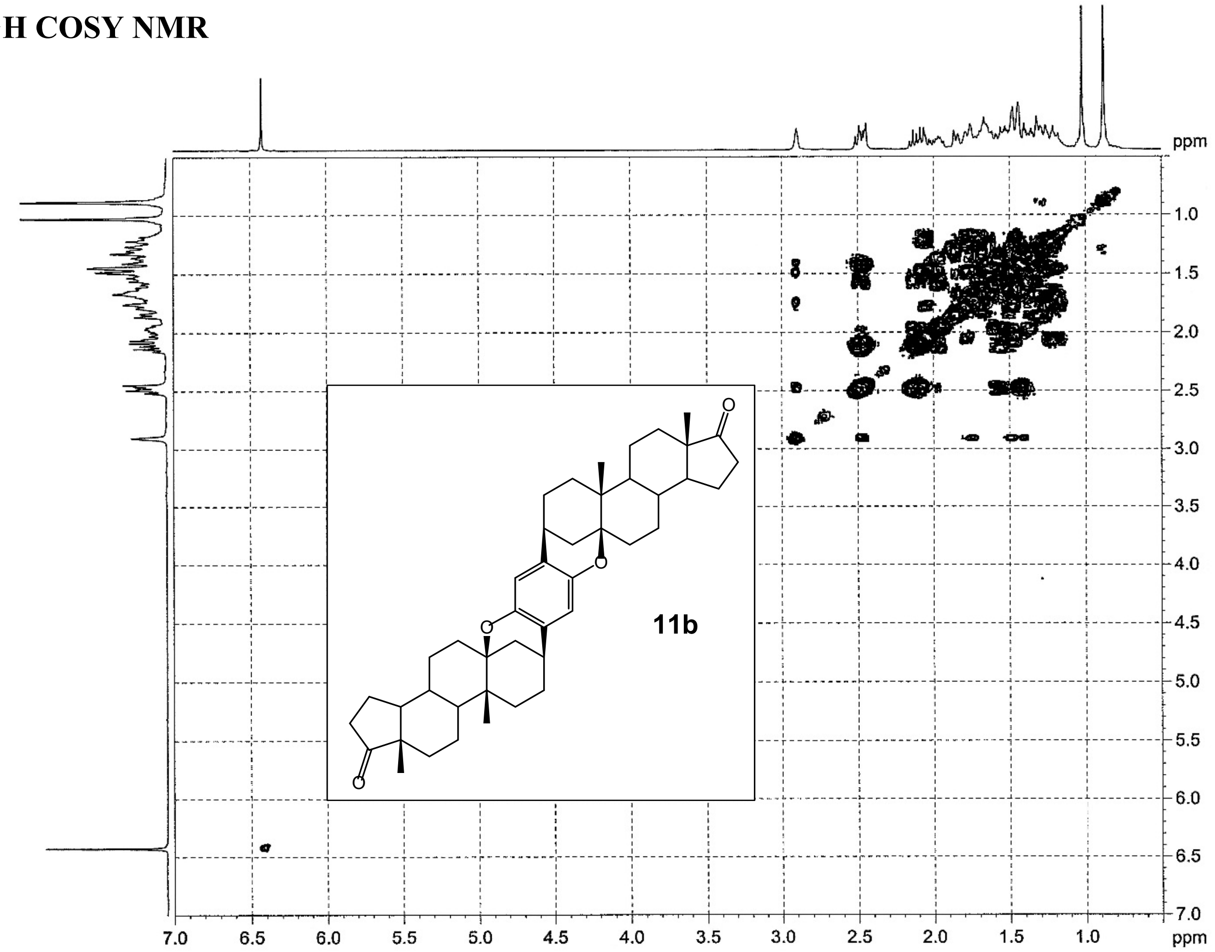

^{1}H-^{13}C HSQC NMR

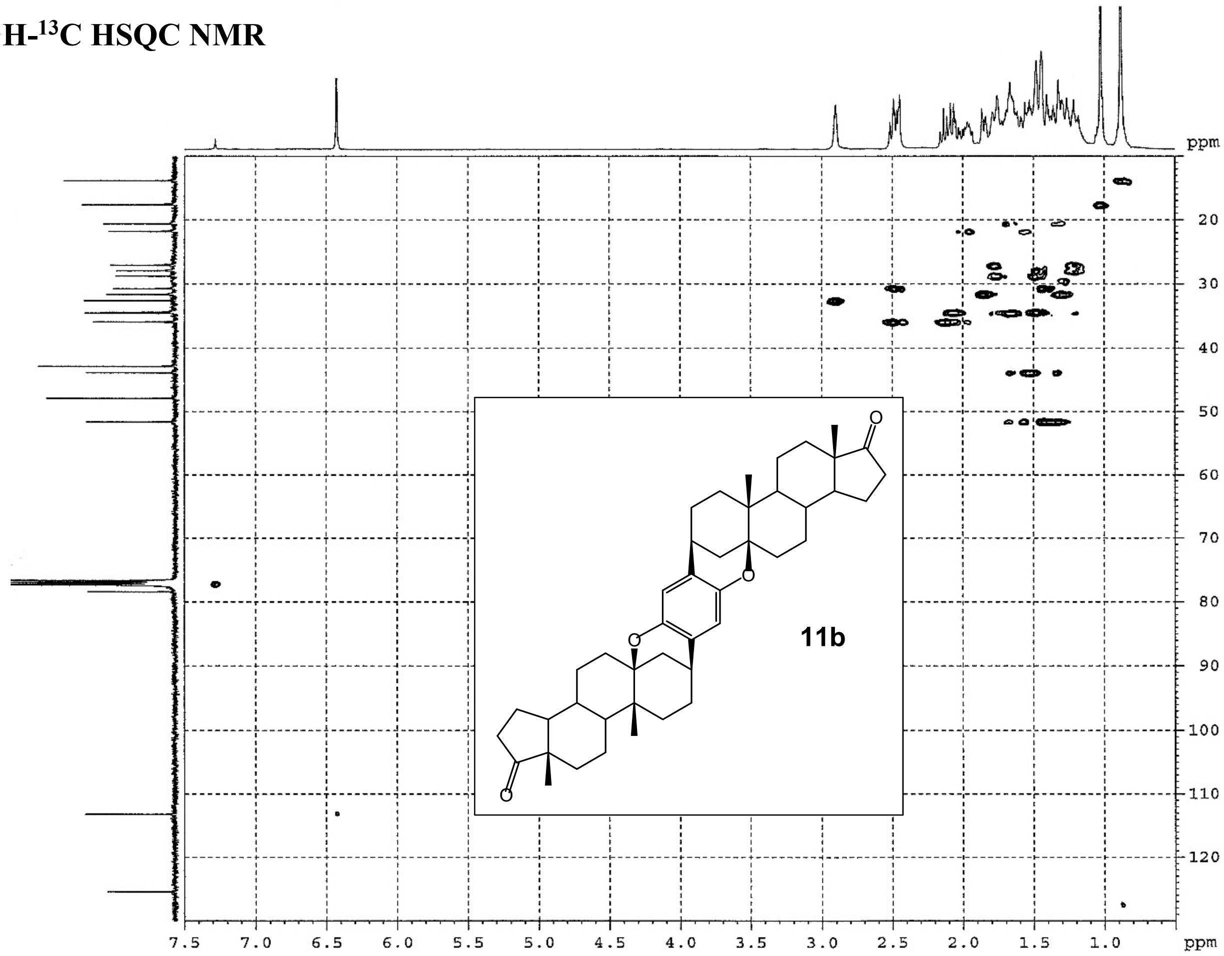

^{1}H-^{13}C HMBC NMR

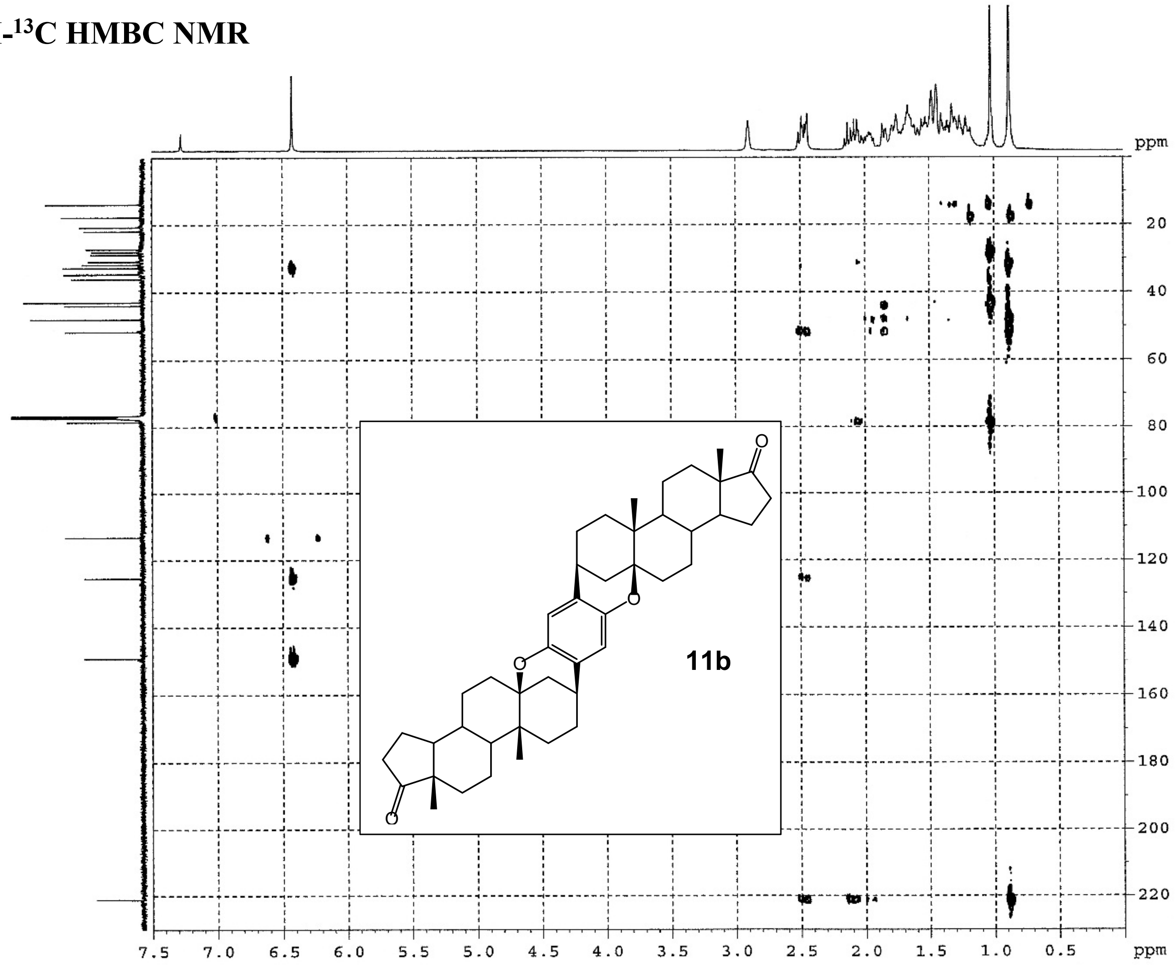

^{1}H-^{13}C HMQC NMR

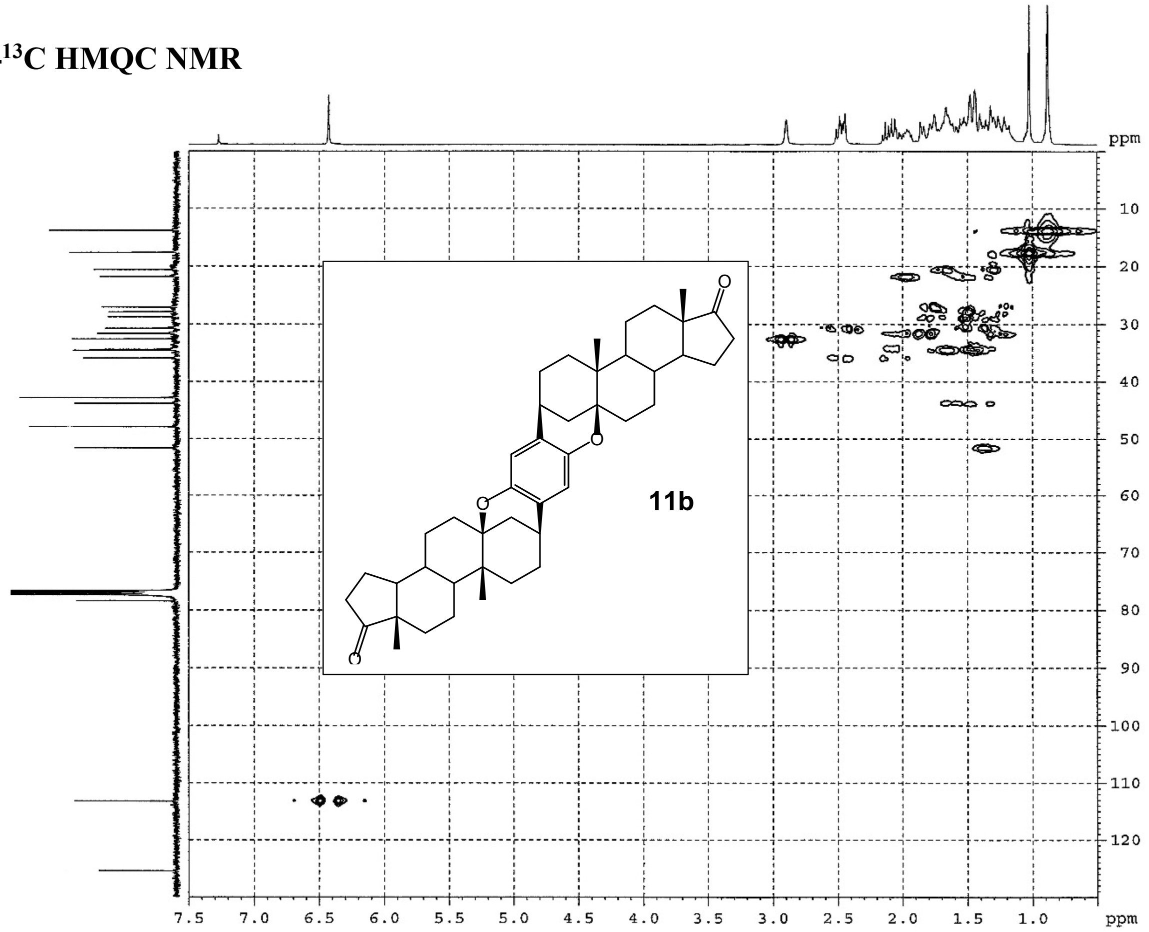

Compound 11c

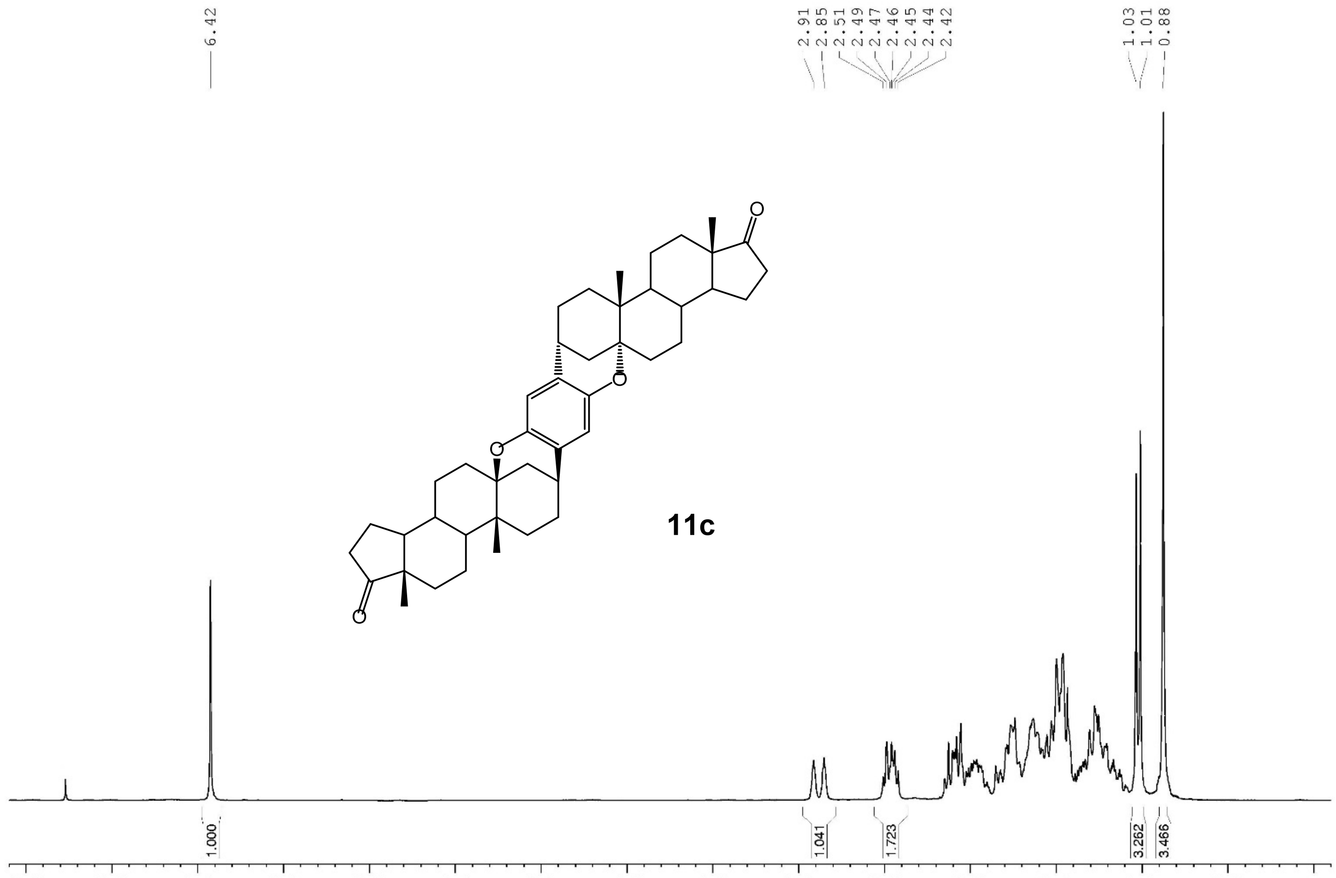

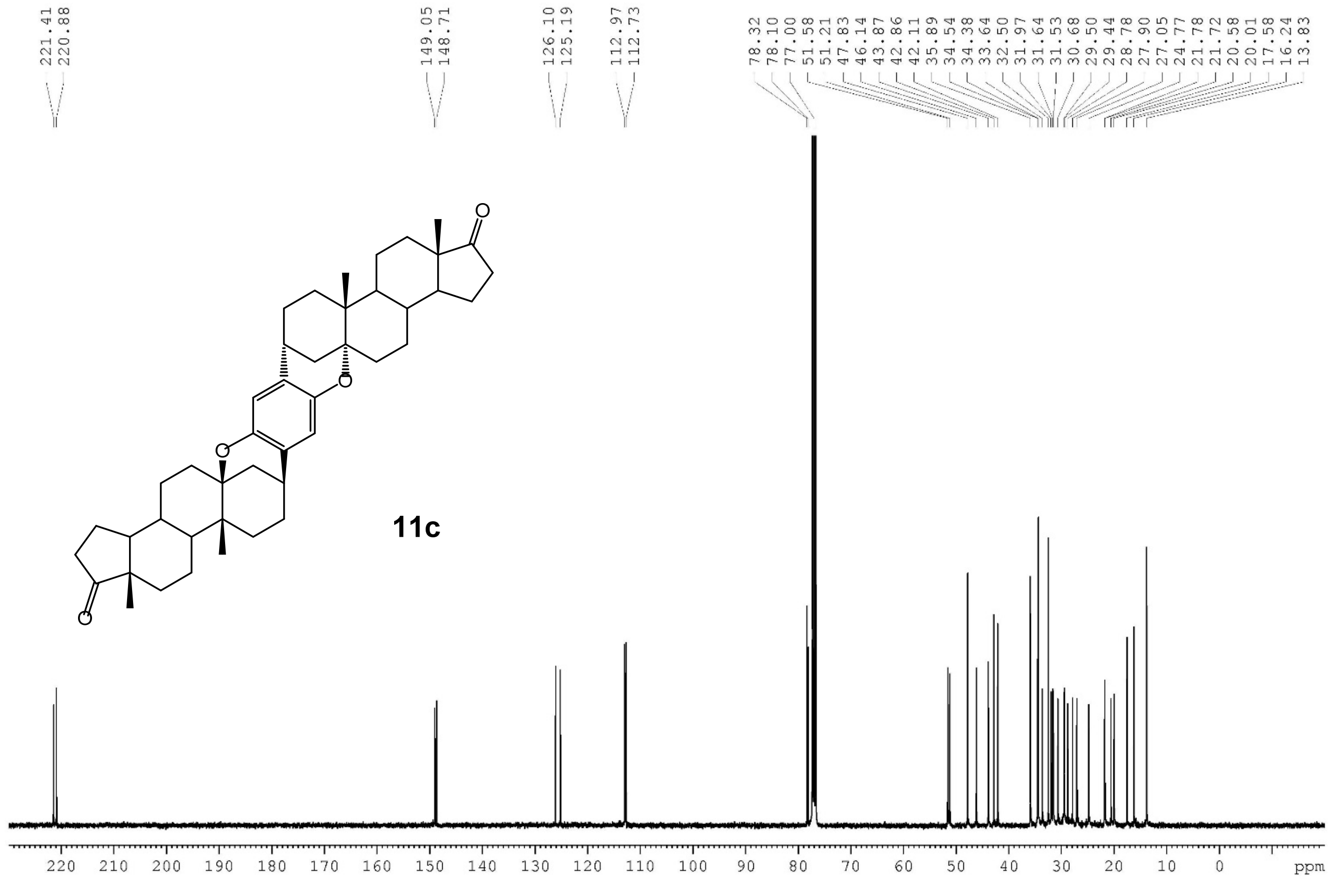

Compound 12

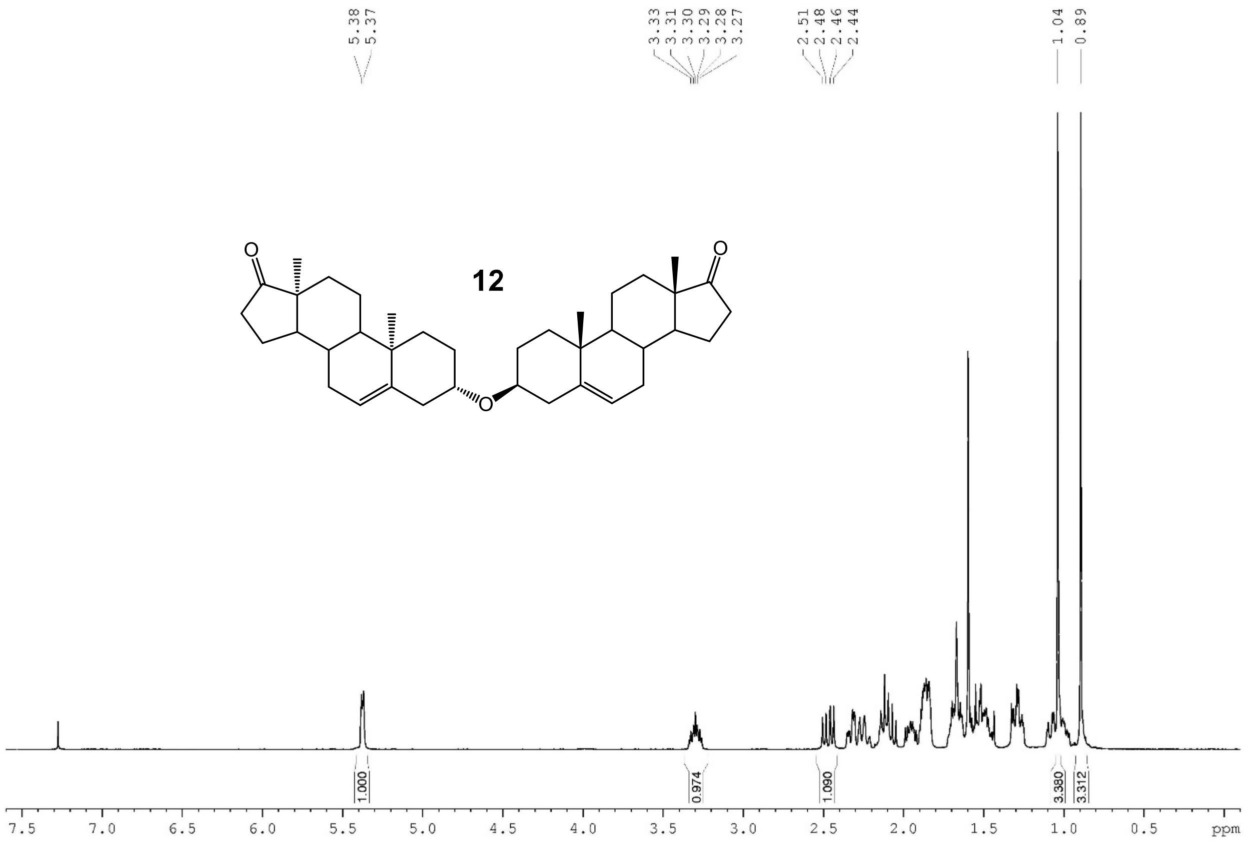
5.38
5.37
3.33
3.31
3.30
3.29
3.28
3.27
2.51
2.48
2.46
2.44
1.04
0.89
12
1.000
0.974
1.090
3.380
3.312
7.5 7.0 6.5 6.0 5.5 5.0 4.5 4.0 3.5 3.0 2.5 2.0 1.5 1.0 0.5 ppm

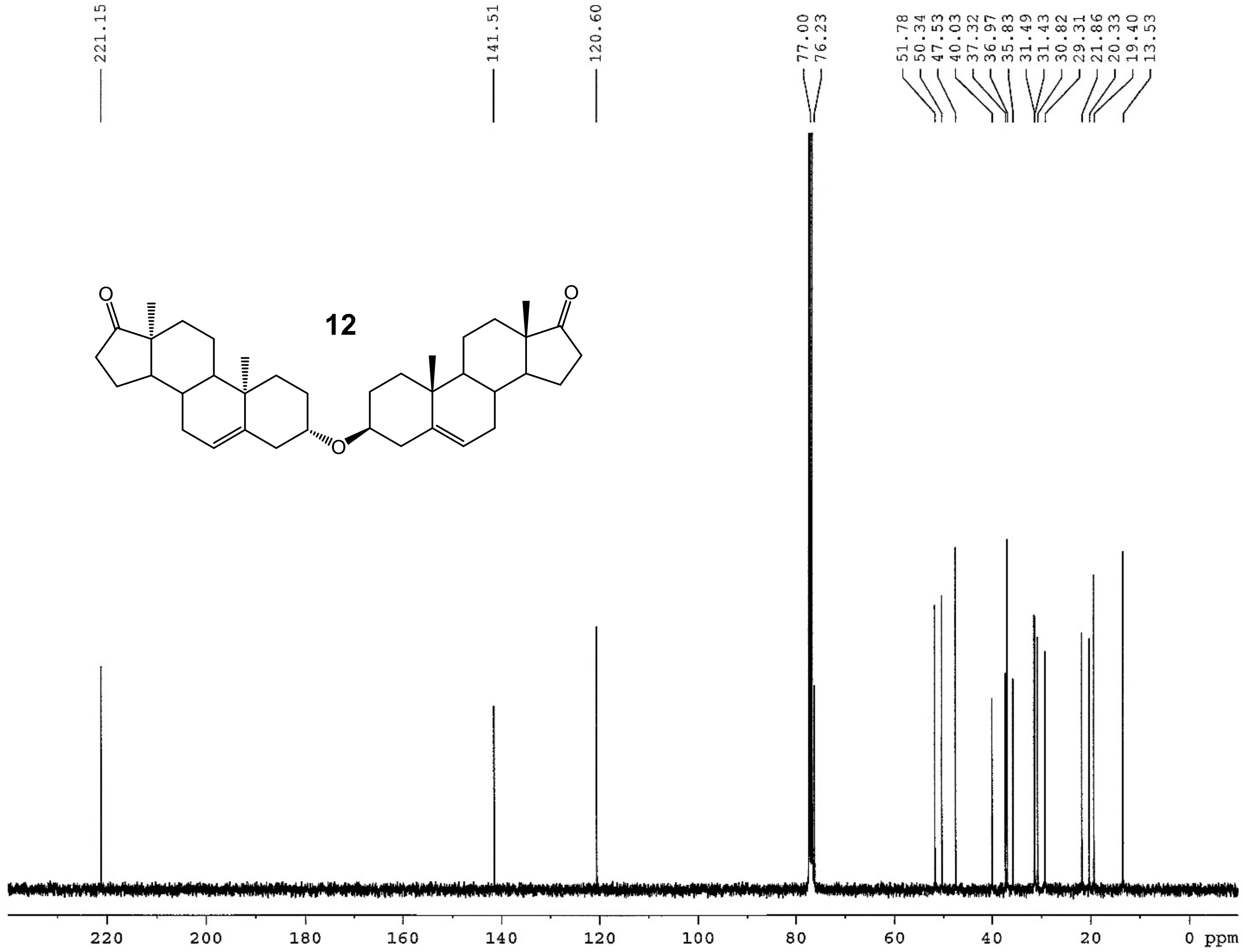
221.15
141.51
120.60
77.00
76.23
51.78
50.34
47.53
40.03
37.32
36.97
35.83
31.49
31.43
30.82
29.31
21.86
20.33
19.40
13.53
12
220 200 180 160 140 120 100 80 60 40 20 0 ppm

Diosgenin derived hydroquinone mono steroidal ether

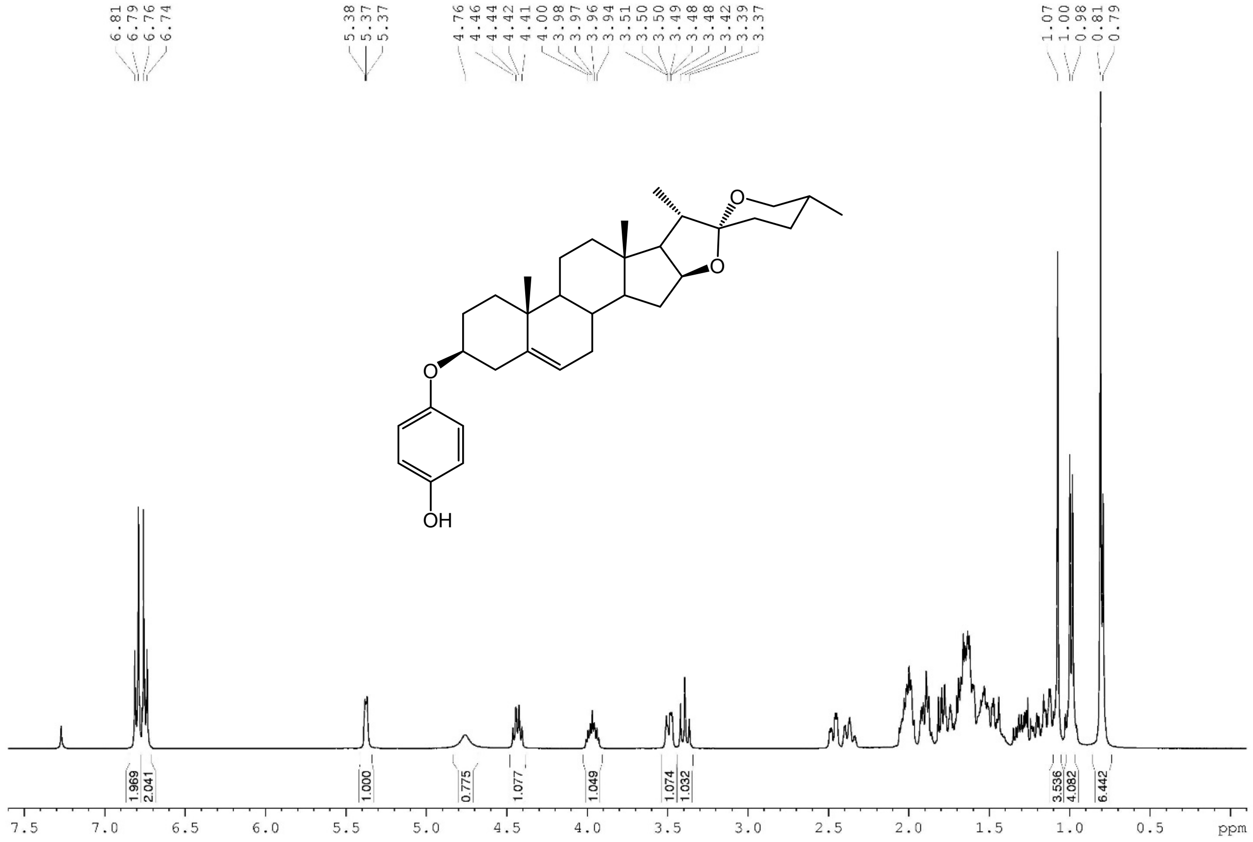

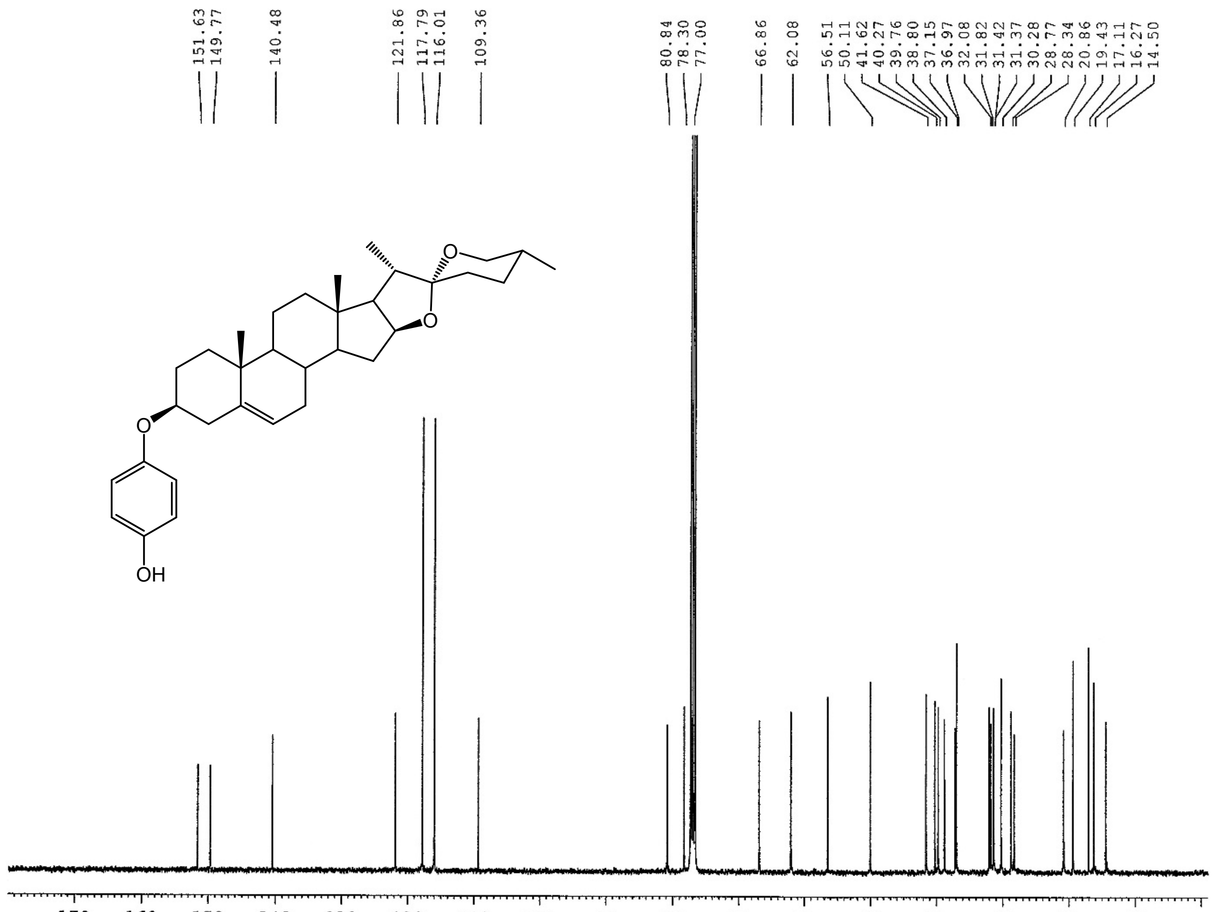

Supplement: Supplementary file 1 [file molecules-28-07068-s001.zip › molecules-2662968-supplementary.pdf]
